# Empirical evidence of declining global vulnerability to climate-related hazards

**DOI:** 10.1016/j.gloenvcha.2019.05.004

**Published:** 2019-07

**Authors:** Giuseppe Formetta, Luc Feyen

**Affiliations:** a Fincons Group, Vimercate, Via Torri Bianche 10, Pal. Betulla, 20871, Vimercate (MB), Italy; b European Commission, Joint European Research Centre (JRC), Ispra, Italy

**Keywords:** Multi-hazard vulnerability, climate related hazard vulnerability

## Abstract

Death tolls and economic losses from natural hazards continue to rise in many parts of the world. With the aim to reduce future impacts from natural disasters it is crucial to understand the variability in space and time of the vulnerability of people and economic assets. In this paper we quantified the temporal dynamics of socio-economic vulnerability, expressed as fatalities over exposed population and losses over exposed GDP, to climate-related hazards between 1980 and 2016. Using a global, spatially explicit framework that integrates population and economic dynamics with one of the most complete natural disaster loss databases we quantified mortality and loss rates across income levels and analyzed their relationship with wealth. Results show a clear decreasing trend in both human and economic vulnerability, with global average mortality and economic loss rates that have dropped by 6.5 and nearly 5 times, respectively, from 1980–1989 to 2007–2016. We further show a clear negative relation between vulnerability and wealth, which is strongest at the lowest income levels. This has led to a convergence in vulnerability between higher and lower income countries. Yet, there is still a considerable climate hazard vulnerability gap between poorer and richer countries.

## Highlights

• We quantified the dynamics of socio-economic vulnerability to climate-related hazards.
• A decreasing trend in both human and economic vulnerability is evident.
• Global average mortality and loss rates have dropped by 6.5 and nearly 5 times, respectively, from 1980 to 1989 to 2007–2016.
• Results also show a clear negative relation between vulnerability and wealth.

## 1 Introduction

Natural hazards continue to cause increasing damage and loss of life. Natural disaster costs globally reached US$314 billion dollars in 2017, more than double the yearly average cost over 2007–2016 (CRED, 2018). Key drivers behind rising losses are exposure changes in terms of rising population and capital at risk (Bouwer, 2011; Visser et al., 2014), as well as better reporting (Paprotny et al., 2018), whereas evidence is growing that anthropogenic climate change is modifying weather and climate extremes (e.g. Donat et al., 2016; Spinoni et al., 2017). Recent independent studies project a further increase of climate hazard impacts in the future connected to anthropogenic warming and socio-economic drivers (e.g. Bouwer, 2013; Winsemius et al., 2016; Dottori et al., 2018; Forzieri et al., 2018; Vousdoukas et al., 2018a, 2018b).

Concurrently, with the Sendai Framework for Disaster Risk Reduction 2015–2030 (UNGA, 2015), the Sustainable Development Goals (UNISDR, 2015) and the Paris Agreement on Climate Change (UNFCCC, 2015), international agreements on disaster loss reduction, development and climate action were recently signed. Disaster reduction, sustainable development and climate change are closely interconnected. Repeatedly, disasters have undermined or made void decade-long poverty reduction efforts, especially in non-industrialized countries (Mysiak et al., 2016), while the poorest countries will likely be affected strongest by rising climate-related disaster risk in a warmer world (Harrington et al., 2018). The Sendai Framework therefore advocates coherence between and mutual reinforcement of policy decisions, monitoring mechanisms and implementation arrangements aimed at reducing disaster risks.

The Sendai Framework further calls for a multi-sectoral, multi-disciplinary and preventive disaster risk reduction strategy, which goes beyond the traditional single hazard, response focused approach. It sets as first priority for action the understanding of disaster risk in all its dimensions. Disaster risk is the combination of three crucial components: i) hazard: natural processes that may causes loss of life, health impacts, property damages and environmental degradation; ii) exposure: human, economic, or environmental assets located in hazard prone areas; and iii) vulnerability: the susceptibility of people, economic/environmental assets to the impacts of hazards (UNISDR, 2009).

Modelling the hazard component is an advanced research activity, with ever improved process understanding, model conceptualizations and parameterizations, spatial coverage and detail. Many studies have analyzed historical trends in hazards based on observations (e.g. Douglas et al., 2000; Hannaford and Marsh, 2006), reanalysis data (e.g. Zolina et al., 2004; Jolly et al., 2015; Schemm et al., 2017) or statistics at national, regional or global scale (e.g., Kundzewicz et al., 2013; Turco et al., 2016).

Recently, much effort has been devoted to create spatially explicit datasets for the dynamic quantification of exposure, such as population, gross domestic product and land-use, from country (e.g. Jongman et al., 2014) to continental (Paprotny et al., 2018) and global scale (Geiger et al., 2018a,2018b; EC, 2015a; Kummu et al., 2018). The maps, although often limited in temporal resolution (most of them available every 5–10 years) and sometimes in spatial resolution (usually between 1 and 50 km), are spatially explicit and provide an added value for understanding trends in natural disaster risk. In the last decade, the explosion of earth observation (EO) data from space is providing more detailed information for quantifying communities’ exposure to natural hazards (Geiß and Taubenböck, 2017). Methods for data collection, merging and processing algorithms have advanced and high resolution satellite images are used to provide global maps of population density (EC, 2015a), urban areas (Esch et al., 2013), and the built environment (Gong et al., 2013). Recent advances in modeling exposure to natural hazards include the use of the new technological paradigm of Big Data (e.g. Yu et al., 2018) and volunteered geographic information systems (e.g. Haworth and Bruce, 2015). The former includes user-generated geo-localized quasi real-time information from micro-blogs (e.g. Twitter, Facebook, Flickr, Instagram), whereas the latter is based on sharing information through crowd-sourcing (e.g. Horita et al., 2015; Cinnamon et al., 2016). Yet, no sufficiently long time series are available from these novel techniques for trend analysis.

Disasters occur when the hazard component interacts with vulnerable exposed population, infrastructure, ecosystems and economic activities. Vulnerability can be defined as the predisposition to incur losses, hence it is the component that has the potential to transform a natural hazard in a disaster. In this sense it is often referred to as the “missing link” (Mechler and Bouwer, 2015; de Brito et al., 2018) for understanding and eventually project climate risks in the future. Vulnerability, including all the actions aimed to reduce the impacts of natural hazards, is dynamic in space and time, is hazard-specific, and depends on environmental, economic, and social factors.

Being a key uncertainty in the disaster risk equation, there is growing interest in understanding and quantifying vulnerability and its dynamics. To date, few studies have analyzed trends in vulnerability at continental to global scale. Jongman et al. (2015) and Tanoue et al. (2016) assessed global river flood vulnerability dynamics by combining high resolution modeling of flood hazard and exposure and demonstrated a general decreasing trend in time of vulnerability. Bouwer and Jonkman (2018) report decreasing mortality rates caused by storm surges at global scale, and also human vulnerability to heat waves in developed countries shows a declining trend (Sheridan and Allen, 2018). Whereas an increasing number of studies attempt to understand present human and economic vulnerability to other hazards (see e.g. Tánago et al. (2016) for an overview on drought vulnerability), typically they are carried out at subnational level (i.e. region, state, or river basin) and dynamics in vulnerability are not well addressed (Jurgilevich et al., 2017).

In this paper we assess the temporal dynamics in the last three decades of human and economic vulnerability to weather-related disasters in a global, multi-hazard, spatially explicit framework. In agreement with other studies that have analyzed natural disaster losses (e.g., Neumayer and Barthel, 2011; Bouwer, 2011; Jongman et al., 2015; Tanoue et al., 2016; Bouwer and Jonkman, 2018; Su et al., 2018), we express vulnerability by mortality rates (reported fatalities as a percentage of exposed population) and loss rates (reported losses as a percentage of exposed GDP). We further investigate the relationship between vulnerability and wealth. Trends in impacts are based on records from Munich RE’s NatCatSERVICE (Munich RE, 2018), one of the most complete natural disaster databases available. Dynamics in exposure are derived from the most recent spatially explicit time-variant population and GDP global maps (EC, 2015a; Geiger et al., 2018b). We quantify the exposed population and GDP based on a neighborhood of the geo-referenced reported event location and perform a sensitivity analysis on the parameter to define this area.

## 2 Materials and methods

Vulnerability (V) describes the relationship between the exposure to a weather-related hazard and the impact. It is analyzed in this study in terms of effects on population (people killed by the weather-related hazard) and economy (monetary losses caused by the hazard). The vulnerability of population is quantified as “mortality rate” (Jongman et al., 2015; Peduzzi et al., 2012), i.e. the ratio between the people killed (R_fat_) by a climate disaster and the people exposed to the hazard (R_p-exp_). Similarly, for economic losses the “loss rate” is used (Jongman et al., 2015), which is the ratio between the economic loss (R_loss_, converted in US$-PPP at the time of the event) caused by the climate disaster and the Gross Domestic Product (GDP, converted in US$-PPP at the time of the event) exposed to the hazard (R_gdp-exp_). We note that GDP may not fully correspond to the wealth stock exposed to disasters. However, due to the absence of good measures of wealth we use GDP as a proxy for wealth, similar to other studies (e.g., Neumayer and Barthel, 2011; Jongman et al., 2015; Tanoue et al., 2016). Assuming mortality and economic loss rates as an indicator of vulnerability is based upon the hypothesis that the rates are higher in more vulnerable regions than in less vulnerable regions.

For the period 1980–2016 we have analyzed the seven weather-related hazards listed in Appendix A, Table A.1: general floods, flash floods, coastal floods, cold related hazard, heatwaves, droughts, and wind related hazards. Data on reported fatalities and direct losses caused by natural disasters in the analyzed period were obtained from Munich RE’s NatCatSERVICE database. This includes the date, the impact (fatalities and official reported economic losses), the type/subtype of the natural disaster, the geo-reference (latitude and longitude) of the center of impact and a description of the event. The section on ‘Disaster database and hazard classification’ in Appendix A provides more information on the dataset and on how we have assigned the events and their impacts to the seven hazard classes analyzed.

The affected area of a given event is not reported in NatCatSERVICE and it is very difficult to delineate for each disaster. Neumayer and Barthel (2011) defined the affected area as a square with size of 100 km × 100 km around the reported georeferenced centroid. We apply a similar method using a circle around the center of impact. In order to assess the influence of the size of the estimated area exposed we perform a sensitivity analysis using four different values for the radius (50, 100, 200, and 400 km). Low values are typically more suitable for localized hazards such as flash floods and wind storms whereas a higher radius better reflects spatially more extensive hazards such as droughts and heatwaves. In the absence of detailed information on the actual affected area, the simplification of using a circle with arbitrary radius may introduce bias in the estimated affected area, and consequently in the absolute mortality and loss rates. However, as this error is likely to be random, with no systematic relatively more under- or overestimating of the true affected area in earlier or later periods (Neumayer and Barthel, 2011), it should not have a significant impact on the trend in vulnerability.

Exposed population and GDP at the time of the event have been derived from the Global Human Settlement Layer (GHSL, EC, 2015a; Pesaresi et al., 2013) population maps and the world-wide spatially explicit GDP maps presented in Geiger (2018b). GHSL provides spatially detailed estimates of the population at 1 km resolution for the target years 1975, 1990, 2000 and 2015. The GDP maps were originally available in 10-years increments between 1850 and 2100 at 5 arcmin resolution. We filled the gaps in time for the analyzed period (1980–2016) by linearly interpolating the population and GDP maps between target years, assuming a constant population and GDP growth rate in between.

For each reported event in the NatCatSERVICE database we overlaid the circle centered in the georeferenced centroid and a fixed radius (in turn 50, 100, 200 and 400 km) with the population and the GDP maps for the year in which the disaster occurred. We then aggregated the grid values within the circle to obtain the location-specific R_p-exp_ and R_gdp-exp_. For general and coastal floods we further masked the population and GDP exposure maps with the 100 year return period respective global scale flood inundated maps (Dottori et al. (2016), https://data.jrc.ec.europa.eu/collection/id-0054; and Vousdoukas et al. (2018a, 2018b), respectively). In this way, within the circle of interest, only people and economic assets within river and coastal flood plains are considered.

The resulting mortality and loss rates are presented by income groups based on the present day World Bank classification (https://datahelpdesk.worldbank.org/knowledgebase/articles/378834-how-does-the-world-bank-classify-countries). We defined two income groups: i) low/middle low (which includes the World Bank low and lower middle income categories) and ii) high/middle high (which include the upper middle and high income categories). This choice allowed us to have representative samples of reported events for all hazards. Especially in the case of droughts and heatwaves, fewer events per year are reported compared to for example flood or wind related hazards.

Finally, we analyzed the relationships between mortality (and loss) rates and wealth for single and multi-hazards. For each reported event we linked the mortality (and loss) rates and the GDP per capita in PPP of the country in the year in which the event occurred. We then binned the GDP per capita in 15 equally-sized classes according its 15 quantiles and reported the average values of GDP per capita and mortality (loss) rates for each class.

## 3 Results

**Fig. 1:**
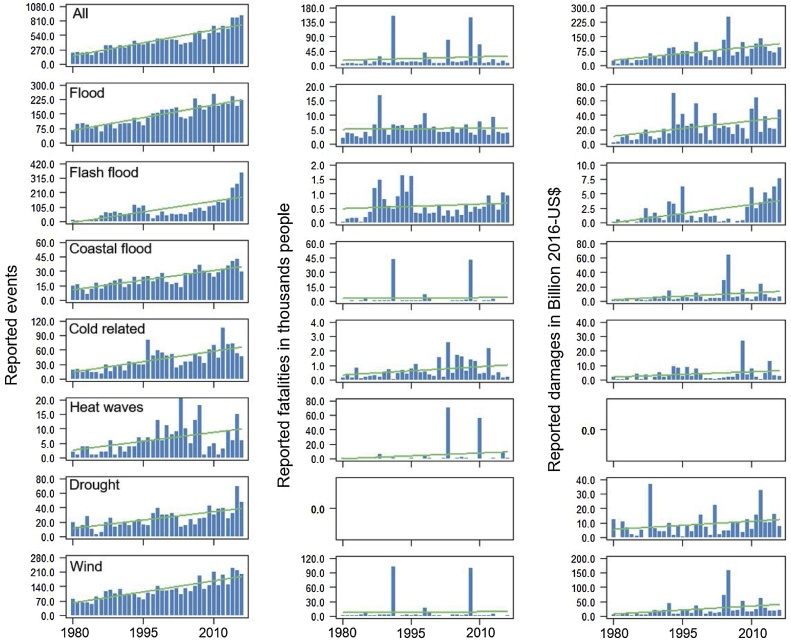
Evolution in time of the reported events, fatalities, and damages occurred between 1980 and 2016. In red is reported the trend line.

For the 7 climate-related hazards considered, the number of events recorded in NatCatSERVICE over the analyzed period (1980–2016) is 16,412. The total reported fatalities amount to 815,293 and total damages to 2,562 billion US$2016 (see Appendix A, Table A.2). The most events are reported for floods (5275) and wind (4570), while for heatwaves only 231 events are recorded. Wind-related disasters are the most lethal and account for nearly 40% of the total fatalities, while wind-related and general flood hazard each represent about one third of the total economic losses. In NatCatSERVICE there are no drought events for which fatalities are reported, whereas the number of heat waves with reported economic losses is less than 30 (Fig. 1). We therefore only look at human vulnerability for heat waves and at economic vulnerability for drought.

The number of reported events and economic impacts (deflated but not normalized with respect to exposed wealth of the year of the event) show a statistically significant increasing trend in the analyzed period both for the 7 hazards together and for each individual hazard (see Table 1 and Fig. 1).

**Table 1:** Summary of the global trend analysis for reported number of events, damages and fatalities. The table reports the variable *G* (reported events, damages and fatalities), the regression coefficient for the year (b), its t and p-value of the regression model G=a+b∙year+ε.

Hazards	Variable	b, year coeff.	t-value	p-value
	Reported events	17 events/year	10.3	***
All	Reported damages	2.6 billion US$2016/year	3.9	***
	*Reported fatalities*	*365 fatalities/year*	*0.71*	
	Reported events	5 events/year	10.3	***
Flood	Reported damages	0.7 billion US$2016/year	3.4	**
	*Reported fatalities*	*12 fatalities/year*	*0.28*	
	Reported events	5 events/year	7.2	***
Flash flood	Reported damages	0.1 billion US$2016/year	3.9	***
	*Reported fatalities*	*6 fatalities/year*	*0.7*	
	Reported events	0.7 events/year	9.2	***
Coastal flood	Reported damages	0.35 billion US$2016/year	1.8	*
	*Reported fatalities*	*24 fatalities/year*	*0.18*	
	Reported events	1.5 events/year	6.3	***
Cold related	*Reported damages*	*0.12 billion US$2016/year*	*2.2*	*
	Reported fatalities	19 fatalities/year	2.4	*
	Reported events	0.2 events/year	3.1	**
Heatwave	Reported damages	–	–	–
	*Reported fatalities*	*270 fatalities/year*	*1.1*	
	Reported events	0.8 events/year	5.3	***
Drought	Reported damages	0.2 billion US$2016/year	2.8	*
	Reported fatalities	–	–	–
	Reported events	4 events/year	9.4	***
Wind	Reported damages	0.9 billion US$2016/year	2.2	*
	*Reported fatalities*	*44 fatalities/year*	*0.1*	

Significance p-value: *** <0.001; ** [0.01-0.001]; * [0.1-0.01]. Variables in italic do not show a statistically significant trend.

The trend in reported fatalities is also increasing but it is not statistically significant. We find a multi-hazard trend in reported (deflated but not normalized) damage of 2.6 billion US$/year (*b* value in Table 1). This is in line with the economic loss growth rate of 3.4 billion US$/year presented in Neumayer and Barthel (2011). The difference is due to the use of a longer time window (1980–2016 vs 1980–2009) and a slightly lower coverage of hazards, as Neumayer and Barthel (2011) include all natural hazards apart from geophysical ones (total sample of 19,360 events) and here only the most relevant climate-related hazards with a sufficiently large sample size to perform a hazard-specific vulnerability analysis are considered (total sample of 16,412). The strongest growth in reported events is observed for flash floods, general floods and wind related hazards, with the latter two also showing the strongest rise in economic losses (growth rate of 0.7 and 0.9 billion US$2016/year, respectively). The smallest rise in the number of reported events is for heatwaves and drought, hence for hazards that occur more sporadically in time.

**Fig. 2:**
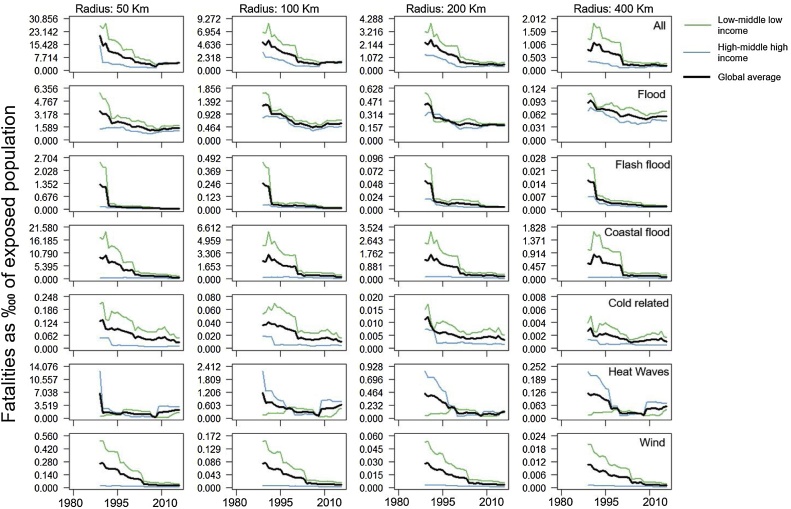
Mortality rates for the analyzed hazards (expressed as number of fatalities per 10 000 people exposed). Results for each hazard represent 10-year moving average of the median (for each year per income class) mortality rates for two income levels (low/middle-low income in green and high/middle-high income in blue) and all countries (average of low/middle-low and high/middle-high income classes). Multi-hazard mortality rates are the sum of single hazard median values.

**Fig. 3:**
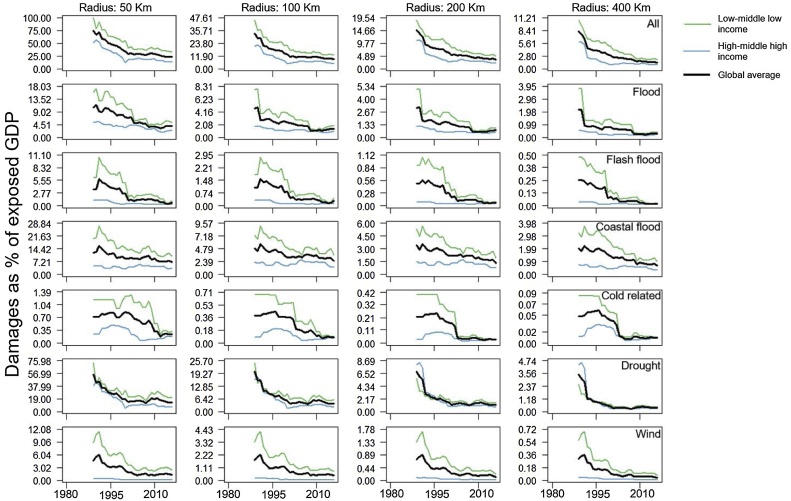
Loss rates for the analyzed hazards. Results for each hazard represent 10-year moving average of the median (for each year per income class) loss rates for two income levels (low/middle-low income in green and high/middle-high income in blue) and all countries (average of low/middle-low and high/middle-high income classes). Multi-hazard loss rates are the sum of single hazard median values.

Over the analyzed period and based on the 7 most common climate-related hazards considered herein, global multi-hazard human (Fig. 2) and economic (Fig. 3) vulnerability show a declining trend across all the radii. From 1980–1989 to 2007–2016, the 10-year moving average mortality rate, averaged over all hazards, radii and both income groups, has reduced more than 6-fold, while the economic loss rate dropped by nearly five times (Table B.1 and B.2 in Appendix). The reduction in vulnerability is stronger earlier in the analyzed period and levels off with time. Further, vulnerability converges between lower and higher income countries due to the stronger vulnerability reduction in less developed countries.

These general trends can also be observed for the individual hazards, and this for the different radii of influence considered. There are, however, a few exceptions. The most notable one is that human vulnerability to heatwaves seems higher in high/middle high income countries. This could be related to several issues with reporting heat mortality, particularly in low income countries, such as non-uniform reporting conventions, not accurate reporting of some causes of deaths, or incomplete information on death certificates (Gall et al., 2009; Mathers et al., 2005; Sehdev and Hutchins, 2001; Azhar et al., 2014). Similarly, poor reporting in developing countries of drought damages likely explains the low economic loss rates, especially prior to 1995-2000. The decreasing trends in hazard vulnerability confirms previous findings at global and regional scales for river floods (Huang, 2013; Jongman et al., 2015; Tanoue et al., 2016), storm surges (Bouwer and Jonkman, 2018), heat waves (Sheridan and Allen, 2018), and winds (Paul et al., 2018).

Notwithstanding the convergence in time of the vulnerability between lower and higher income countries for the analyzed hazards, the present (10-year average over 2007–2016) multi-hazard mortality rate is still 4.4 times larger in low/middle low income countries (see Table B.2, Appendix B). Hence, over the last four decades the difference in multi-hazard human vulnerability between poorer and richer countries reduced by almost 2.5 times (see Table B.1 in Appendix B). The present gap in human vulnerability varies strongly between hazards. For low/middle-low income countries, hazard vulnerabilities are higher by a factor ranging from 1.5 for general floods up to 9 for coastal floods and wind related hazards. For heatwaves, reported fatalities suggest higher human vulnerability in high/middle-high income countries. Yet, as previously stated, this can likely be attributed to under-reporting of heat mortality, especially in low income countries.

Economic loss rates show similar behavior in time, with patterns also consistent across the different radii analyzed. The 10-year average 2007–2016 multi-hazard economic loss rate is almost four times higher in lower income countries compared to higher income countries (see Table B.3, Appendix B). This is about halve compared to the period 1980–1989 (see Table B.4, Appendix B). The present gap in economic vulnerability for single hazards ranges between 1.4 for cold related hazards to around 10 for wind related hazards. Coastal floods, flash floods, droughts, and general floods show a factor difference of 3.2, 2.3, 2.3, and 2.2 respectively (see Table B.3, Appendix B). Jongman et al. (2015) found a factor difference between high and low income countries of 17 for mortality and 3 for losses caused by floods at the end of the 2000s. These values are higher compared to 1.5 for mortality and 2.2 for losses that we find for general floods. This is in part because the values of Jongman et al. (2015) reflect the difference between low- and high-income countries from the four-class World Bank classification (low-, lower middle, upper middle-, and high-income categories). Differently from Jongman et al. (2015) our classification is based two income groups: low/middle low (which includes the low- and lower middle categories) and high/middle high (which includes the upper middle- and high-income categories). Moreover, the fact that we analyze a different time period partly explains the discrepancy with results of Jongman et al. (2015).

**Fig. 4:**
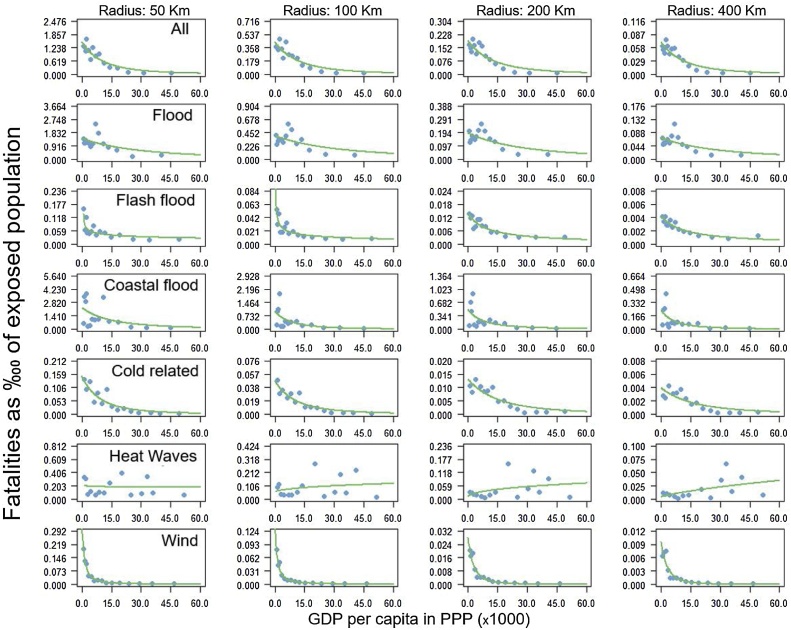
Mortality rates as function of the wealth for multi and single hazards. Mortality rates are expressed as number of fatalities per 10 000 people exposed. Wealth is approximated by the GDP per capita (in US$-PPP) at the time of the event.

**Fig. 5:**
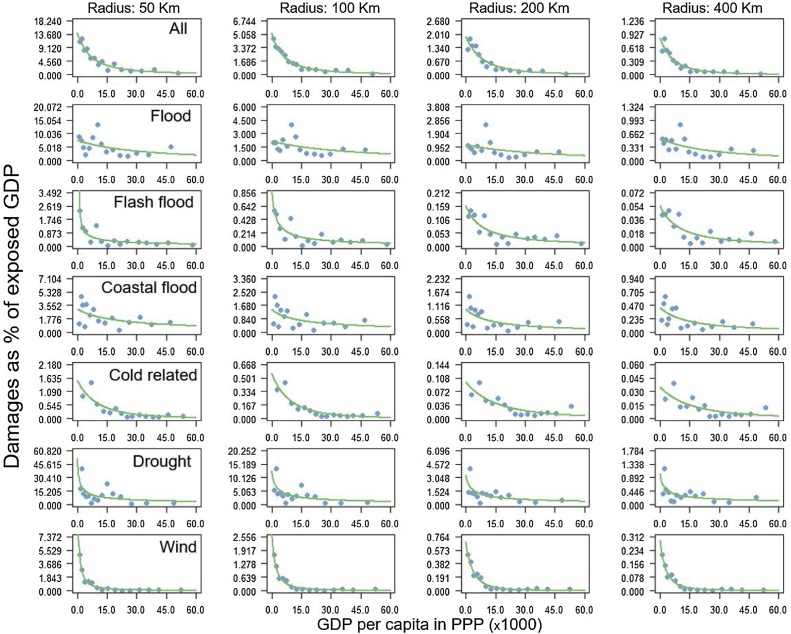
Loss rates (in US$-PPP at the time of the event) as function of the wealth for multi and single hazards. Wealth is approximated by the GDP per capita (in US$-PPP) at the time of the event.

There is a clear negative relationship between mortality and loss rates and wealth, here approximated by the GDP per capita in PPP of the year of the event (Fig. 4, Fig. 5, respectively). The latter have been derived from figures C.1 and C.3 in Appendix C, through the binning procedure described in Section 2. For better visualization of the trend we fitted a power law function through the data with non-linear regression. In all cases the parameters of the functions showed a statistically significant p-value (all p-values<0.1).

The decline in vulnerability with increasing wealth is consistent across the radii analyzed. It is strongest for the lowest ranges of GDP per capita and weakens as income levels become higher. This holds both for human and for economic vulnerability. The reduction in mortality and loss rates with increasing wealth is evident both for the multi-hazard analysis as well as for the single hazards, apart from heatwave mortality. This confirms previous findings for multiple hazards (e.g. Toya and Skidmore, 2007; Kahn, 2005) and for specific hazards (e.g. Jongman et al., 2015 and Tanoue et al., 2016 for floods). The patterns described for Fig. 4, Fig. 5 can also be observed in the raw data (Figs. C1-C2 in Appendix C). These further show the high variability in mortality and loss rates across GDP per capita for the different hazards, indicating that there is large uncertainty around the smoothed curves obtained after binning.

For floods, the fitted monotonically decreasing trend line does not align well with the data for the lowest income ranges. It can be argued that the nonlinear relationship between mortality (loss) rate and wealth shows an initial increase before showing a monotonic decrease, as suggested by Kellenberg and Mobarak (2008) and Zhou et al. (2014). The mortality rate for heatwaves does not show a clear relation with wealth. This could be due to under-reporting in lower income countries combined with recent extreme heatwaves events that occurred in high income countries, such as the July-September 2010 Russian heatwave with a total of 56,000 fatalities and the July-August 2003 European heat wave that caused a total of 68,312 fatalities.

## 4 Discussion and conclusions

Understanding vulnerability of our societies to hazards remains a critical hurdle in accurate disaster risk assessments. In this work we presented, to our best knowledge, the first global scale, spatially variable multi-hazard analysis of dynamics in human and economic vulnerability to the most impacting climate hazards. Expressing natural hazard impacts as a share of the exposed population/GDP rather than in absolute terms helps in understanding the greater burden for poorer countries. Although high income countries may suffer higher absolute losses, in lower income countries people and their belonging are less protected and more vulnerable to natural hazards (UNISDR, 2018). Our findings have important implications. Improved protection against hazards has counter-balanced the effects of increasing exposure on disaster risk, with the global average 2007–2016 multi-hazard human mortality and loss rates dropping of about 6.5 and nearly 5 times as compared to the period 1980–1989, respectively. The more a country is developed the higher are the investments in protection measures to natural hazards, early warning systems, and disaster risk management strategies. These actions facilitate not only the response but also the recovering phase that follow a natural disaster (e.g. Cavallo and Noy, 2010). This is confirmed by the clear negative relation between vulnerability and wealth for all the analyzed hazard except heatwaves. This effect is strongest at lower income levels and diminishes with increasing wealth, which has resulted in a reduction of the vulnerability gap between higher and lower income countries because lower income countries have adapted relatively faster compared to higher income countries. Nevertheless, a considerable vulnerability gap between low and high income countries is still evident for specific hazards such as coastal floods and wind related mortality rates (factor of 9) and for wind related loss rate (factor of 10). This suggests that poorer countries remain particularly vulnerable to these hazards and that huge investments or changes in these societies may be needed to further reduce their vulnerability to them. For example, implementing and maintaining coastal protection measures can be very costly and may only be achievable when a certain level of wealth is attained. In many lower income tropical and subtropical countries with coasts, mangroves have also declined rapidly as they are cleared for coastal development and aquaculture and logged for timber and fuel production (Polidoro et al., 2010), counteracting efforts to reduce coastal flood risk. Wind-related hazards are less confined in space compared to for example river and flash floods, and reducing their impacts requires changes in building and infrastructure standards over extended domains.

Carrying out the vulnerability analysis by grouping countries in two income classes (namely high and low income countries) averages loss and mortality rate differences between countries classified as high and medium-high income, and low and medium low income, respectively. For floods these differences have been found marked (e.g., Jongman et al., 2015). The subdivision of countries in two broad income groups was adopted to include all hazard types in a common vulnerability analysis framework. For hazards such as heatwaves and droughts the sample of events with reported impacts were not sufficiently large to build vulnerability functions using a country classification based on four income categories.

Understanding vulnerability is hampered by the availability of harmonized and reliable data of human, environmental and economic losses. It is widely acknowledged that NatCatSERVICE is one of the most comprehensive global disaster loss databases available. Like most of the global/regional publicly available or proprietary databases (e.g. EM-DAT, DesInventar, Swiss Re’s Sigma) it suffers weaknesses such as under/over/miss reporting of the impacts, gaps in historical records, and bias by high impact events (e.g. Gall et al., 2009; Gall, 2015). Events having limited time-space context, so called invisible or neglected events (e.g. Zaidi, 2018; Wisner and Gaillard, 2009; Khan and Kelman, 2012) remain largely unobserved and unreported and constitute an additional source of underestimation of the impacts.

NatCatSERVICE is a database owned by Munich RE, which primary interest is to understand insured losses. In order to verify potential bias in the data towards insured losses in richer countries, a comparison with EM-DAT in terms of number of events, fatalities and losses is presented in Figures D1-D6 in Appendix D. The events classification in EM-DAT has been done following the methodology presented for NatCatSERVICE (see Appendix A). We note that less than 5% of EM-DAT events could not be classified because lack of information on the event type. The comparison shows that the number of reported events is in general larger in NatCatSERVICE, especially for higher income countries. The total number of fatalities across all hazards is very similar between databases for both income groups. Total losses seem in general somewhat higher in NatCatSERVICE for the higher income countries, whereas there is no consistent difference between the databases across the time period for lower income countries. The most notable differences for certain hazards (e.g., wind/coastal and floods/flash floods) likely relate to a different categorization of some events in these classes in the respective databases. Moreover, losses from localized events such as flash floods or winds often are insured and reported by insurance companies but not necessarily appear in (international) newspapers and thus in EM-DAT.

Most disaster databases only include estimates of direct losses that are immediately visible after the occurrence of the event. Indirect losses that may occur in the aftermath of the event, such as loss of jobs or business interruption, as well as consequential losses visible months or years after the disaster, such as reduced country GDP and lower currency exchange rate, are not typically documented (e.g. Wirtz et al., 2014; Gall, 2015). These impacts can vary strongly and most estimates of their magnitude are based on modeling rather than empirical analysis (Kousky, 2014). Further, apart from the fatalities, people can suffer a wide range of impacts from disasters, often with delayed effects (e.g., Schmitt et al., 2016). Hence, human and economic vulnerability go beyond mortality and direct economic loss rate considered herein.

In order to achieve progress in reaching the disaster risk reduction targets of the Sendai Framework and implementing the Sustainable Development Goals, there is a need for a well-defined, accurate, standardized and systematic procedure to collect disaster impacts, especially at the local level. Evaluations of damage and risk mitigation costs should be fed into national and international open-access databases to improve the evidence basis for better understanding vulnerability and decision making to reduce it (Kreibich et al., 2014). The UN Office for Disaster Risk Reduction (UNISDR) has therefore stepped up efforts to improve the collection of data on disaster losses. In March 2018 it launched the Sendai Framework Monitor (SFM), an online tool designed to capture data on achieving the Sendai targets. By October 2018, already 61 countries have started using the SFM and report mainly on four targets for disaster losses: mortality, numbers of people affected, economic losses and damage to critical infrastructure.

Another critical issue for understanding vulnerability is the exact delineation of the area exposed to damaging intensities of the hazard. It is unique for each hazard event and it may vary considerably among disasters of the same hazard type. For example, wind related hazards can act very local (e.g. tornado) or induce damages over extended domains (e.g. tropical cyclone). We show that the trend in vulnerability vs time and wealth is not strongly affected by the delineation of the area exposed, as the shapes of the functions are consistently decreasing across the analyzed radii. However, for risk assessments it is important to accurately quantify vulnerability (i.e. mortality and loss rates) in order to more reliably translate the exposed people, assets and wealth into human and economic loss estimates. Hence, reporting of disaster losses should also include a better delineation and mapping of the exact area affected.

Finally, there is need for spatially explicit information on socio-economic drivers of vulnerability and impacts. We used GDP as a proxy for the wealth exposed and this may lead to biased estimates of the actual stock exposed. Especially when economies become more service-oriented (developed countries) this may overemphasize loss reductions in these countries in recent years. This could be overcome by using for example information on capital stock, yet this information is not available globally at the relevant temporal and spatial resolution. Further, we show a clear negative relation between GDP per capita and vulnerability, yet the latter depends on several other factors, such as aid dependency, inequality, education level, infrastructure, health status and size of the financial sector (e.g., Toya and Skidmore, 2007). More research is needed to understand and quantify the contribution of these drivers of vulnerability.

## References

[bib0005] Azhar G.S., Mavalankar D., Nori-Sarma A., Rajiva A., Dutta P., Jaiswal A., Sheffield P., Knowlton K., Hess J.J. (2014). Heat-related mortality in India: excess all-cause mortality associated with the 2010 Ahmedabad heat wave. PLoS One.

[bib0010] Bouwer L.M. (2011). Have disaster losses increased due to anthropogenic climate change?. Bull. Am. Meteorol. Soc..

[bib0015] Bouwer L.M. (2013). Projections of future extreme weather losses under changes in climate and exposure. Risk Anal..

[bib0020] Bouwer Laurens M., Jonkman Sebastiaan N. (2018). Global mortality from storm surges is decreasing. Environ. Res. Lett..

[bib0025] Cavallo E., Noy I. (2010). The Economics of Natural Disasters: a Survey. IDB Working Paper Series:124. http://www.iadb.org/en/research-and-data/publication-details,3169.html?pub_id=idb-wp-124.

[bib0030] Cinnamon J., Jones S.K., Adger W.N. (2016). Evidence and future potential of mobile phone data for disease disaster management. Geoforum.

[bib0035] de Brito M.M., Evers M., Almoradie S., Delos A. (2018). Participatory flood vulnerability assessment: a multi-criteria approach. Hydrol. Earth Syst. Sci..

[bib0040] Donat M.G., Alexander L.V., Herold N., Dittus A.J. (2016). Temperature and precipitation extremes in century-long gridded observations, reanalyses, and atmospheric model simulations. J. Geophys. Res..

[bib0045] Dottori F., Salamon P., Bianchi A., Alfieri L., Hirpa F.A., Feyen L. (2016). Development and evaluation of a framework for global flood hazard mapping. Adv. Water Resour..

[bib0050] Dottori F., Szewczyk W., Ciscar J.-C., Zhao F., Alfieri L., Hirabayashi Y., Bianchi A., Mongelli I., Frieler K., Betts R.A., Feyen L. (2018). Increased human and economic losses from river flooding with anthropogenic warming. Nat. Clim. Change.

[bib0055] Douglas E.M., Vogel R.M., Kroll C.N. (2000). Trends in floods and low flows in the United States: impact of spatial correlation. J. Hydrol..

[bib0060] EC (2015). Joint Research Centre (JRC); Columbia university, Center for International Earth Science Information Network - CIESIN (2015): GHS Population Grid, Derived from GPW4, Multitemporal. http://data.europa.eu/89h/jrc-ghsl-ghs_pop_gpw4_globe_r2015a.

[bib0065] Esch T., Marconcini M., Felbier A., Roth A., Heldens W., Huber M., Schwinger M., Taubenböck H., Müller A., Dech S. (2013). Urban footprint processor—fully automated processing chain generating settlement masks from global data of the TanDEM-X mission. IEEE Geosci. Remote Sens. Lett..

[bib0070] Forzieri G., Bianchi A., Silva F.B.E., Marin Herrera M.A., Leblois A., Lavalle C., Aerts J.C.J.H., Feyen L. (2018). Escalating impacts of climate extremes on critical infrastructures in Europe. Glob. Environ. Chang. Part A.

[bib0075] Gall M., Borden K.A., Cutter S.L. (2009). When do losses count? Six fallacies of natural hazards loss data. Bull. Am. Meteorol. Soc..

[bib0080] Gall (2015). Melanie. "The Suitability of Disaster Loss Databases to Measure Loss and Damage from Climate Change." International Journal of Global Warming 8.2.

[bib0085] Geiger T., Frieler K., Bresch D.N. (2018). A global historical data set of tropical cyclone exposure (TCE-DAT). Earth Syst. Sci. Data.

[bib0090] Geiger T. (2018). Continuous national gross domestic product (GDP) time series for 195 countries: past observations (1850–2005) harmonized with future projections according to the shared socio-economic pathways (2006–2100). Earth Syst. Sci. Data.

[bib0095] Geiß C., Taubenböck H. (2017). One Step Back for a Leap Forward: Toward Operational Measurements of Elements at Risk.

[bib0100] Gong P., Wang J., Yu L., Zhao Y.C., Zhao Y.Y., Liang L., Niu Z., Huang X., Fu H., Liu S. (2013). Finer resolution observation and monitoring of global land cover: first mapping results with Landsat TM and ETM+ data. Int. J. Remote Sens..

[bib0105] Hannaford Jamie, Marsh Terry (2006). An assessment of trends in UK runoff and low flows using a network of undisturbed catchments. Int. J. Climatol..

[bib0110] Harrington L.J., Frame D., King A.D., Otto F.E.L. (2018). How uneven are changes to impact-relevant climate hazards in a 1.5 °C world and beyond?. Geophys. Res. Lett..

[bib0115] Haworth B., Bruce E. (2015). A review of volunteered geographic information for disaster management. Geogr. Compass.

[bib0120] Horita F.E., de Albuquerque J.P., Degrossi L.C., Mendiondo E.M., Ueyama J. (2015). Development of a spatial decision support system for flood risk management in Brazil that combines volunteered geographic information with wireless sensor networks. Comput. Geosci..

[bib0125] Huang G. (2013). Does a Kuznets curve apply to flood fatality? A holistic study for China and Japan. Nat Hazards.

[bib0130] Jolly W.M., Cochrane M.A., Freeborn P.H., Holden Z.A., Brown T.J., Williamson G.J., Bowman D.M.J.S. (2015). Climate-induced variations in global wildfire danger from 1979 to 2013. Nat. Commun..

[bib0135] Jongman B., Koks E.E., Husby T.G., Ward P. (2014). J.: Increasing flood exposure in the Netherlands: implications for risk financing. Nat. Hazards Earth Syst. Sci..

[bib0140] Jongman B., Winsemius H.C., Aerts J.C., de Perez E.C., van Aalst M.K., Kron W., Ward P.J. (2015). Declining vulnerability to river floods and the global benefits of adaptation. Proc. Natl. Acad. Sci..

[bib0145] Jurgilevich A., Räsänen A., Groundstroem F., Juhola S. (2017). A systematic review of dynamics in climate risk and vulnerability assessments. Environ. Res. Lett..

[bib0150] Kahn M.E. (2005). The death toll from natural disasters: the role of income, geography, and institutions. Rev. Econ. Stat..

[bib0155] Khan Shabana, Kelman Ilan (2012). Progressive climate change and disasters: connections and metrics. Nat. Hazards.

[bib0160] Kellenberg D.K., Mobarak A.M. (2008). Does rising income increase or decrease damage risk from natural disasters?. J. Urban Econ..

[bib0165] Kreibich H., Van Den Bergh J.C.J.M., Bouwer L.M., Bubeck P., Ciavola P., Green C., Hallegatte S., Logar I., Meyer V., Schwarze R., Thieken A.H. (2014). Costing natural hazards. Nat. Clim. Change.

[bib0170] Kummu M., Taka M., Guillaume J.H. (2018). Gridded global datasets for gross domestic product and Human Development Index over 1990–2015. Sci. Data.

[bib0175] Kousky C. (2014). Informing climate adaptation: a review of the economic costs of natural disasters. Energy Econ..

[bib0180] Mathers C.D., Ma Fat D., Inoue M., Rao C., Lopez A.D. (2005). Counting the dead and what they died from: an assessment of the global status of cause of death data. Bull. World Health Organ..

[bib0185] Mechler R., Bouwer L.M. (2015). Understanding trends and projections of disaster losses and climate change: is vulnerability the missing link?. Clim. Change.

[bib0190] Munich R.E. (2018). NatCatSERVICE Database (Munich Reinsurance Company, Geo Risks Research, Munich). https://www.munichre.com/en/reinsurance/business/non-life/natcatservice/index.html.

[bib0195] Neumayer E., Barthel F. (2011). Normalizing economic loss from natural disasters: a global analysis. Global Environ. Change.

[bib0200] Kundzewicz Z.W., Pińskwar I., Brakenridge G.R. (2013). Large floods in Europe, 1985-2009. Hydrol. Sci. J..

[bib0205] Mysiak J., Surminski S., Thieken A., Mechler R., Aerts J.C. (2016). Brief communication: Sendai framework for disaster risk reduction–success or warning sign for Paris?. Nat. Hazards Earth Syst. Sci..

[bib0210] Paprotny D., Sebastian A., Morales-Nápoles O., Jonkman S.N. (2018). Trends in flood losses in Europe over the past 150 years. Nat. Commun..

[bib0215] Paul S.H., Sharif H.O., Crawford A.M. (2018). Fatalities caused by hydrometeorological disasters in Texas. Geosciences.

[bib0220] Peduzzi P., Chatenoux B., Dao H., De Bono A., Herold C., Kossin J., Nordbeck O. (2012). Global trends in tropical cyclone risk. Nat. Clim. Change.

[bib0225] Pesaresi M., Huadong G., Blaes X., Ehrlich D., Ferri S., Gueguen L., Marin-Herrera M.A. (2013). A global human settlement layer from optical HR/VHR RS data: concept and first results. IEEE J. Sel. Top. Appl. Earth Obs. Remote. Sens..

[bib0230] Polidoro B.A., Carpenter K.E., Collins L., Duke N.C., Ellison A.M., Ellison J.C., Livingstone S.R. (2010). The loss of species: mangrove extinction risk and geographic areas of global concern. PLoS One.

[bib0235] Schemm S., Sprenger M., Martius O., Wernli H., Zimmer M. (2017). Increase in the number of extremely strong fronts over Europe? A study based on ERA‐Interim reanalysis (1979–2014). Geophys. Res. Lett..

[bib0240] Sehdev A.E.S., Hutchins G.M. (2001). Problems with proper completion and accuracy of the cause-of-death statement. Arch. Intern. Med..

[bib0245] Sheridan S.C., Allen M.J. (2018). Temporal trends in human vulnerability to excessive heat. Environ. Res. Lett..

[bib0250] Schmitt L.H.M., Graham H.M., White P.C.L. (2016). Economic evaluations of the health impacts of weather-related extreme events: a scoping review. Int. J. Environ. Res. Public Health.

[bib0255] Spinoni J., Naumann G., Vogt J.V. (2017). Pan-European seasonal trends and recent changes of drought frequency and severity. Glob. Planet Change.

[bib0260] Tánago I.G., Urquijo J., Blauhut V., Villarroya F., De Stefano L. (2016). Learning from experience: a systematic review of assessments of vulnerability to drought. Nat. Hazards.

[bib0265] Tanoue M., Hirabayashi Y., Ikeuchi H. (2016). Global-scale river flood vulnerability in the last 50 years. Sci. Rep..

[bib0270] Toya H., Skidmore M. (2007). Economic development and the impacts of natural disasters. Econ. Lett..

[bib0275] Turco M., Bedia J., Di Liberto F., Fiorucci P., Von Hardenberg J., Koutsias N., Llasat M.-C., Xystrakis F., Provenzale A. (2016). Decreasing fires in mediterranean Europe. PLoS One.

[bib0280] UNGA (2015). Sendai Framework for Disaster Risk Reduction 2015–2030.

[bib0285] UNFCCC (2015). Paris Agreement.

[bib0290] UNISDR (2009). United Nations International Strategy for Disaster Reduction (UNISDR). (2009). Terminology on Disaster Risk Reduction. https://www.unisdr.org/we/inform/publications/7817.

[bib0295] UNISDR (2018). Economic Losses, Poverty & DISASTERS. https://www.unisdr.org/2016/iddr/CRED_Economic%20Losses_10oct_final.pdf.

[bib0300] Visser H., Petersen A.C., Ligtvoet W. (2014). Climatic Change.

[bib0305] Vousdoukas M.I., Mentaschi L., Voukouvalas E., Bianchi A., Dottori F., Feyen L. (2018). Climatic and socioeconomic controls of future coastal flood risk in Europe. Nat. Clim. Change.

[bib0310] Vousdoukas M.I., Mentaschi L., Voukouvalas E., Verlaan M., Jevrejeva S., Jackson L.P., Feyen L. (2018). Global probabilistic projections of extreme sea levels show intensification of coastal flood hazard. Nat. Commun..

[bib0315] Winsemius H.C., Aerts J.C.J.H., Van Beek L.P.H., Bierkens M.F.P., Bouwman A., Jongman B., Kwadijk J.C.J., Ligtvoet W., Lucas P.L., Van Vuuren D.P., Ward P.J. (2016). Global drivers of future river flood risk. Nat. Clim. Change.

[bib0320] Wirtz A., Kron W., Löw P., Steuer M. (2014). The need for data: natural disasters and the challenges of database management. Nat. Hazards.

[bib0325] Wisner B., Gaillard J.C. (2009). An introduction to neglected disasters. Jàmbá: J. Disaster Risk Stud..

[bib0330] Yu M., Yang C., Li Y. (2018). Big data in natural disaster management: a review. Geosciences.

[bib0335] Zaidi R.Z. (2018). Beyond the Sendai indicators: application of a cascading risk lens for the improvement of loss data indicators for slow-onset hazards and small-scale disasters. Int. J. Disaster Risk Reduct..

[bib0340] Zhou Y. (2014). Socioeconomic development and the impact of natural disasters: some empirical evidences from China. Nat Hazards.

[bib0345] Zolina O., Kapala A., Simmer C., Gulev S.K. (2004). Analysis of extreme precipitation over Europe from different reanalyses: a comparative assessment. Glob. Planet Change.

