# Supplementary material for: Empirical evidence of declining global vulnerability to climate-related hazards
Source: Glob Environ Change. 2019 Jul;57:101920. doi: 10.1016/j.gloenvcha.2019.05.004 (PMC6686205; doi:10.1016/j.gloenvcha.2019.05.004)
Supplement: Supplementary file 1 [file mmc1.docx]

**Appendix A**

Disaster database and hazard classification

We analyzed the seven natural hazards listed in Table A.1: general floods, flash floods, coastal floods, cold related hazards, heatwaves, droughts, and wind related hazards. The events were classified according to the “Event” field of the NatCatSERVICE database (Primary event type in Table A.1). An obstacle to correctly identify the impacts for coastal floods, wind and cold related hazards is the fact that, depending on their main triggering causes and impacts, they are classified as tropical cyclones (tc:Tropical cyclone), tempests (te:Tempest/Severe storm), or winter storms (ws:Winter storm) in the “Subevent” field of the NatCatSERVICE database (Secondary event type in Table A.1). We have identified these events and associated them to the analyzed hazard as reported in Table A.1. For example, the analyzed hazard “Coastal flood” includes: i) events classified as “ss:Storm surge” in the “Event” field of the NatCatSERVICE database (Primary event type in Table A.1); and ii) some events classified as “tc:Tropical cyclone” in the “Subevent” field of the NatCatSERVICE database (Secondary event type in Table A.1).

The selection of Secondary type events to include for the analyzed hazard is based on the “event description” field available in the NatCatSERVICE database. It contains a description of the occurred event in textual form, specifying the type of damages produced and the affected items. Using the software R-cran and the package “tokenizers” (https://cran.r-project.org/web/packages/tokenizers/tokenizers.pdf) we automatically scrutinized the “event description” field for some specific keywords that refer to the analyzed hazard (Keywords in Table A.1). Secondary type events have been included in the coastal floods, wind and cold related hazards if their description field contains one of the keywords related to the analyzed hazard. Yet, only a portion of the impacts (fatalities and losses) reported for the disaster were assigned to Secondary type events, where the rest of the impacts was assigned to the corresponding Primary events, in order not to double count for impacts. To test the sensitivity of the splitting ratio three values have been selected: 10%, 30%, and 50% of total reported damages of the disaster assigned to the Secondary type event. In the main text, results and figures are based on a 30% vs 70% splitting ratio, with 30% of the damages assigned to the Secondary type event and 70% to the Primary type event. In the appendix results are presented for a splitting ratio of 10% vs 90% and 50% vs 50%. To give a real example, a tropical cyclone occurred in Bangladesh on May 1994 and caused 200 fatalities and economic losses of 255 million 2016-US$ dollar. The description field for this event states: “"Wind gusts up to 200 km/h, heavy rain, storm surge, >52,000 houses/dwellings destroyed and >17,400 damaged. Roads, railroads, bridges damaged. Traffic, train service affected. Trees and power lines downed. Fishing boats sunk. Losses to crops. Injured: >7,000, homeless/evacuated: 500,000.” In this case we assigned 30% (10% and 50%) of the impacts (100 fatalities and 127.5 million 2016-US$) to a coastal flood event, and 70% to wind related hazards (tropical cyclone).

Table A.2 presents the total number of reported events, fatalities and damages (in Billion US$2016) that occurred world-wide in the analyzed period (1980-2016). Results in brackets show the same quantities for the original NatCatSERVICE classification, i.e. before splitting reported disaster damages over co-occurring hazards. Coastal flooding is the hazard most affected by the re-classification: the number of reported events increases from 100 to 835, fatalities from 461 to 118,190, and damages from 0.1 to 277.96 billion US$2016. For wind related hazards the number of events increases by including those from “te:Tempest/Severe storm”, yet the overall impacts decrease as part of the impacts of events classified as ‘tc:Tropical cyclone” is re-assigned to coastal floods impacts. Finally, floods, flash floods, droughts and heatwaves remain un-changed with respected to the original NatCatSERVICE database. Results for splitting ratios of 10% vs 90% and 50% vs 50% show similar patterns both in terms of total damages/fatalities (see Table A.3 and A.4, respectively) and the vulnerability curves (see Figures A.1 to A.4).

Table A.1: List of hazards analyzed and criteria used for their classification.

| **Analyzed Hazard** | **Primary event type** | **Secondary event type** | **keywords** |
| --- | --- | --- | --- |
| General flood | general flood (gf) |  |  |
| Flash Flood | flash flood (ff) |  |  |
| Coastal Flood | storm surge (ss) | tropical cyclone (tc) | coast, coastal, coastline, flash, flood, flooded, flooding, floods, inundated, marinas, marine, maritime, overflowed, surge, surges, coast, coastal, coastline, flash, flood, flooded, flooding, floods, inundated, marinas, marine, maritime, overflowed, surge, surges |
| Cold Related | cold wave/frost (cw)  blizzard/snowstorm (bs)  winter damage (wd) | winter storm (ws) | avalanches, blizzards, freezing, frost, hail, ice, icing |
|  |  | tempest/severe storm (te) | ice, snow, snowfall , snowstorm , snowstorms |
| Heat wave | Heatwave (hw) |  |  |
| Drought | Drought (dr) |  |  |
| Wind | local windstorm (lw)  tropical cyclone (tc) | winter storm (ws) | roof, roofs, speed, speeds, tree, tornadoes, tornado, wind, winds, windstorm |
|  |  | tempest/severe storm (te) | wind, speeds, trees, roofs, tornadoes, tornado, winds, plant, roof, tree, unroofed, windstorm |

Table A.2: Total reported events, fatalities and damages in billion US$2016 for the analyzed hazards after classification. In brackets the same quantities reported according to original classification in NatCatSERVICE database.

| Analyzed hazard | Total reported events | Total reported fatalities | Total reported damages (Billion US$2016) |
| --- | --- | --- | --- |
| Flood | 5275 (5275) | 194190 (194190) | 862.78 (862.78) |
| Flash flood | 3111 (3111) | 21095 (21095) | 68.40 (68.40) |
| Coastal flood | 835 (100) | 118190 (461) | 277.96 (0.10) |
| Cold related | 1471 (1171) | 24778 (24019) | 148.05 (127.10) |
| Heatwave | 231 (231) | 160991 (160991) | 27.77 (27.77) |
| Drought | 919 (919) | 0 (0) | 323.86 (323.86) |
| Wind | 4570 (3172) | 296049 (410266) | 853.27 (1019.90) |
| Total | 16412 (13979) | 815293 (811022) | 2562.09 (2429.91) |

Table A.3: Total reported events, fatalities and damages in Billion US$2016 for the analyzed hazards considering a splitting ratio of 10% vs 90%. In brackets the same quantities reported in the original NatCatSERVICE database.

| Analyzed hazard | Total reported events | Total reported fatalities | Total reported damages (Billion US$2016) |
| --- | --- | --- | --- |
| Flood | 5275 (5275) | 194190 (194190) | 862.78 (862.78) |
| Flash flood | 3111 (3111) | 21095 (21095) | 68.40 (68.40) |
| Coastal flood | 835 (100) | 39705 (461) | 92.72 (0.10) |
| Cold related | 1471 (1171) | 24272 (24019) | 134.06 (127.10) |
| Heatwave | 231 (231) | 160991 (160991) | 27.77 (27.77) |
| Drought | 919 (919) | 0 (0) | 323.86 (323.86) |
| Wind | 4570 (3172) | 372193 (410266) | 963.80 (1019.90) |
| Total | 16412 (13979) | 812446 (811022) | 2473.39 (2429.91) |

Table A.4: Total reported events, fatalities and damages in Billion US$2016 for the analyzed hazards considering a splitting ratio of 50% vs 50%. In brackets the same quantities reported in the original NatCatSERVICE database.

| Analyzed hazard | Total reported events | Total reported fatalities | Total reported damages (Billion US$2016) |
| --- | --- | --- | --- |
| Flood | 5275 (5275) | 194190 (194190) | 862.78 (862.78) |
| Flash flood | 3111 (3111) | 21095 (21095) | 68.40 (68.40) |
| Coastal flood | 835 (100) | 196677 (461) | 463.21 (0.10) |
| Cold related | 1471 (1171) | 25285 (24019) | 162.04 (127.10) |
| Heatwave | 231 (231) | 160991 (160991) | 27.77 (27.77) |
| Drought | 919 (919) | 0 (0) | 323.86 (323.86) |
| Wind | 4570 (3172) | 219906 (410266) | 742.74 (1019.90) |
| Total | 16412 (13979) | 818144 (811022) | 2473.39 (2429.91) |


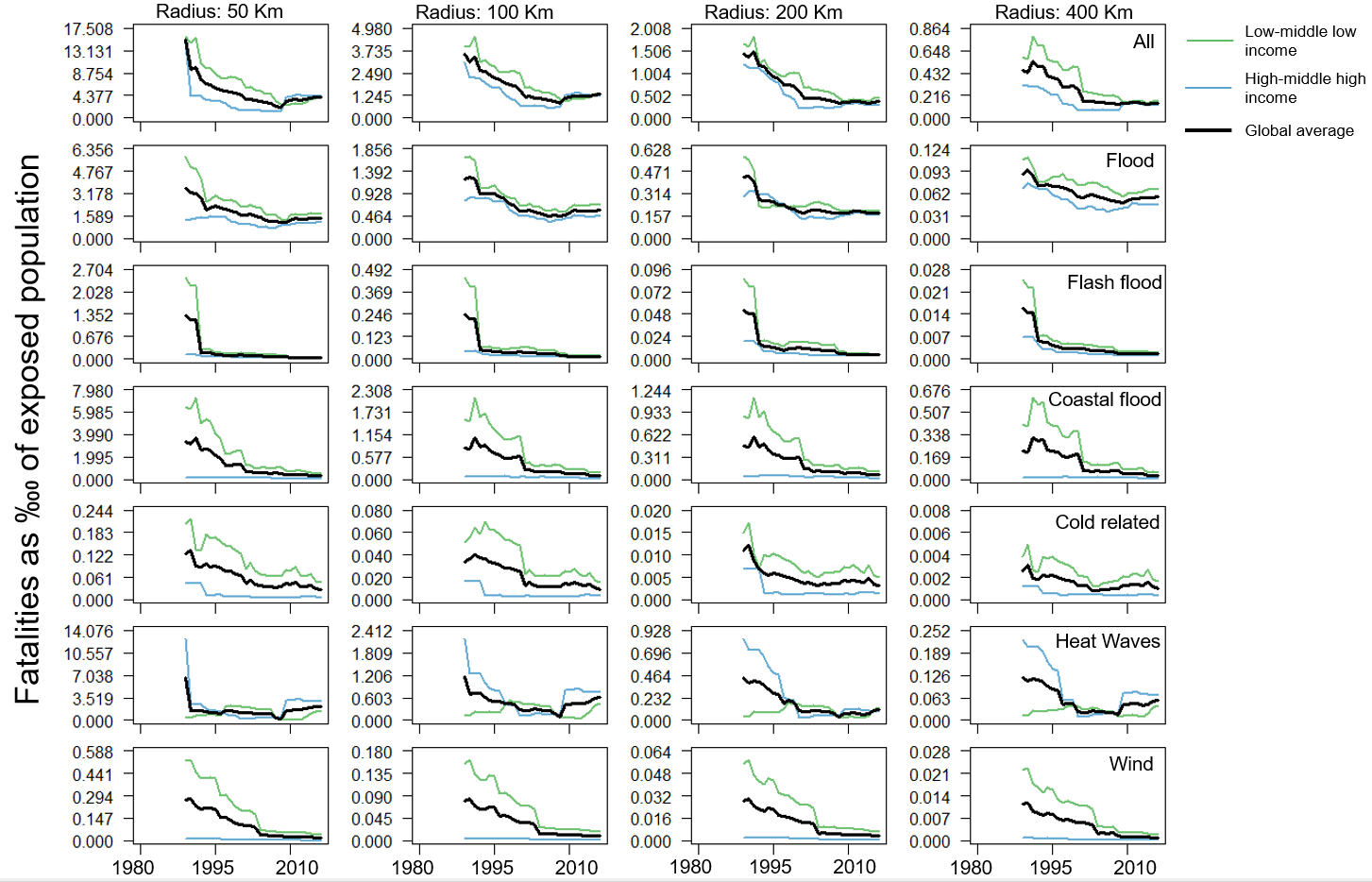


Figure A.1: Mortality rates caused by the analyzed hazards based on splitting ratio 10% vs 90%. Mortality rates are expressed as number of fatalities per 10 000 people exposed. Results are presented by income levels (low income in orange and high income in blue) and as global average (black).


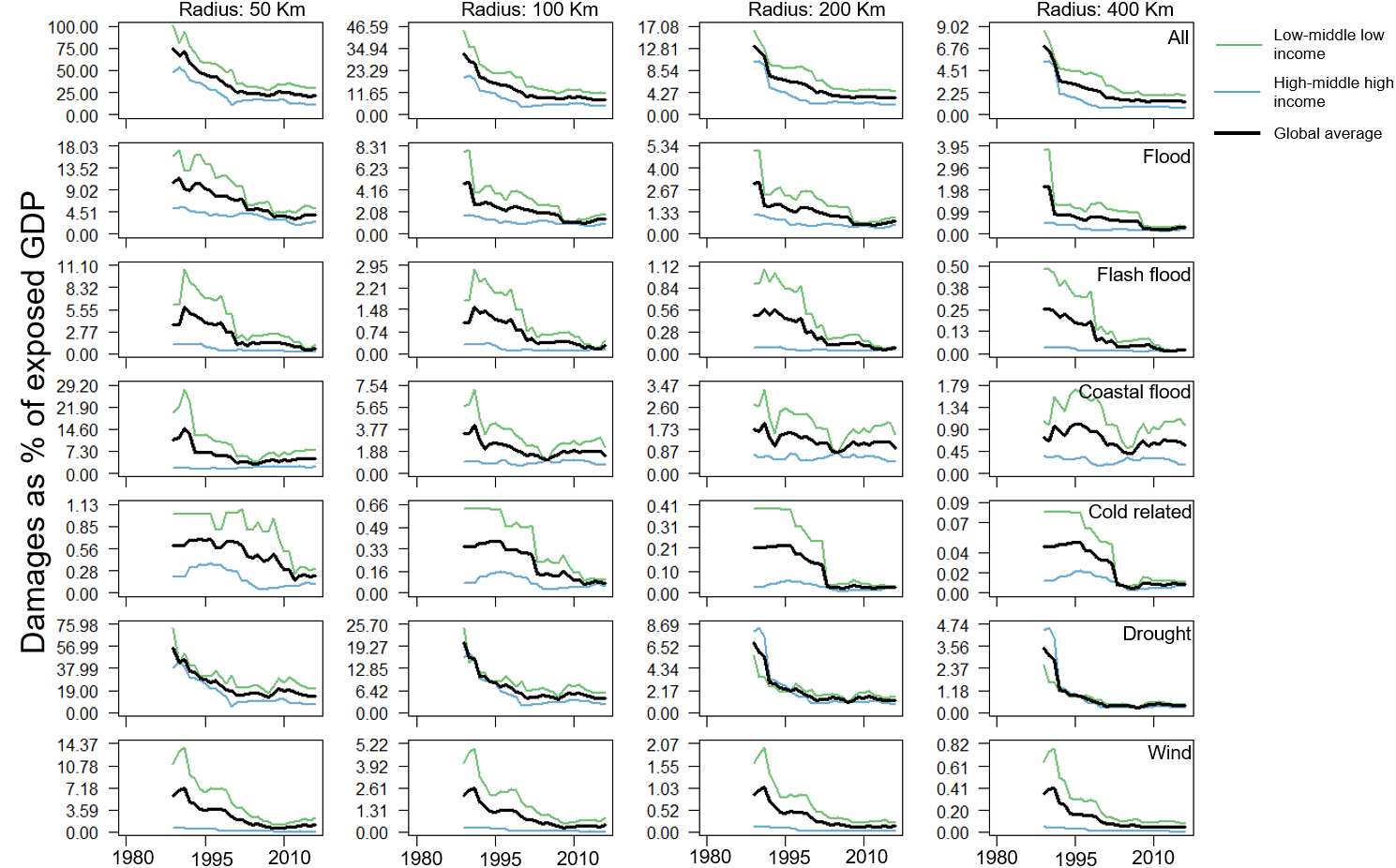


Figure A.2: Loss rates caused by the analyzed hazards based on splitting ratio of 10% vs 90%. Results are presented by income levels (low income in orange and high income in blue) and as global average (black).


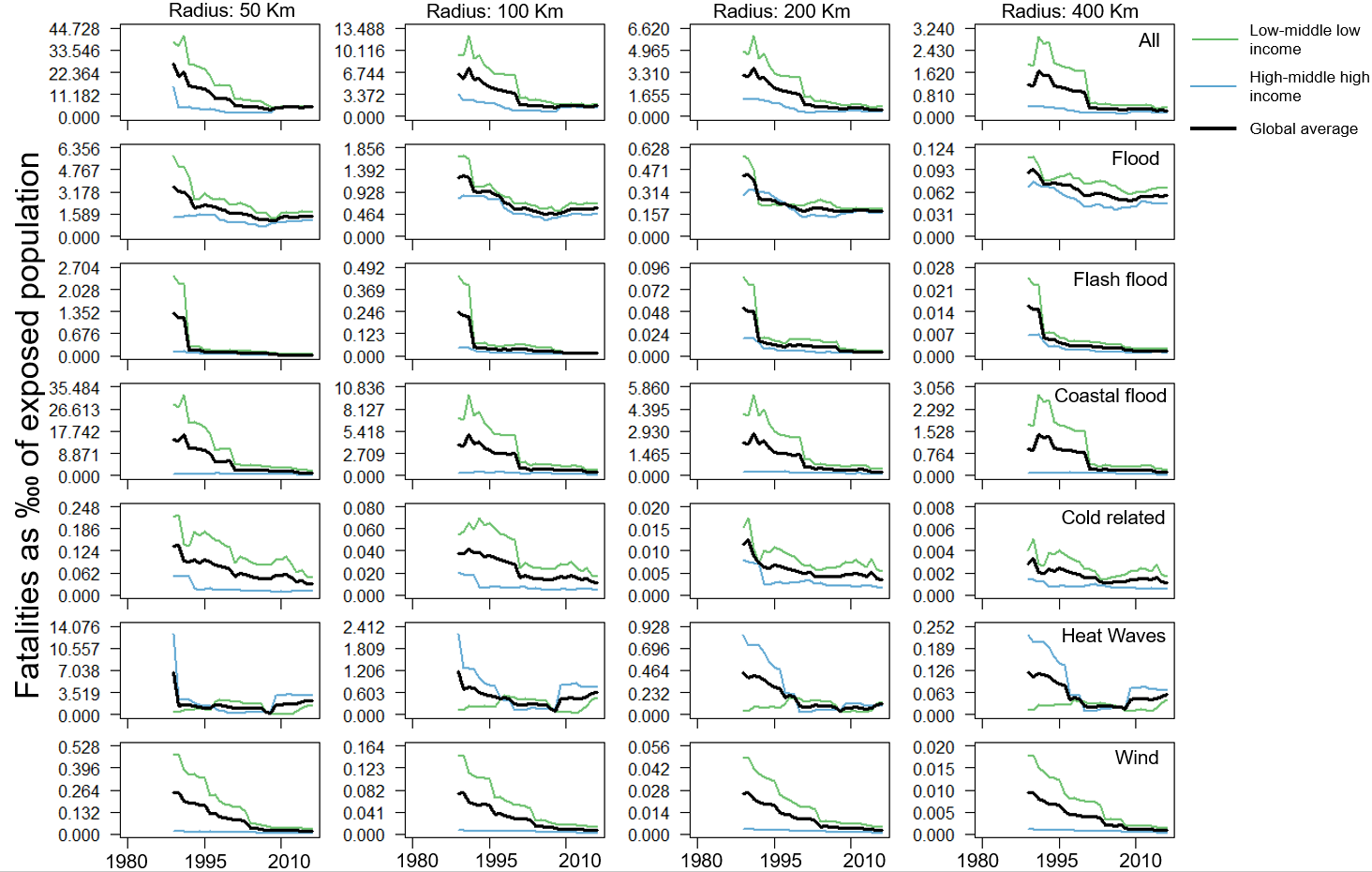


Figure A.3: Mortality rates caused by the analyzed hazards splitting ratio of 50% vs 50%. Mortality rates are expressed as number of fatalities per 10 000 people exposed. Results are presented by income levels (low income in orange and high income in blue) and as global average (black).


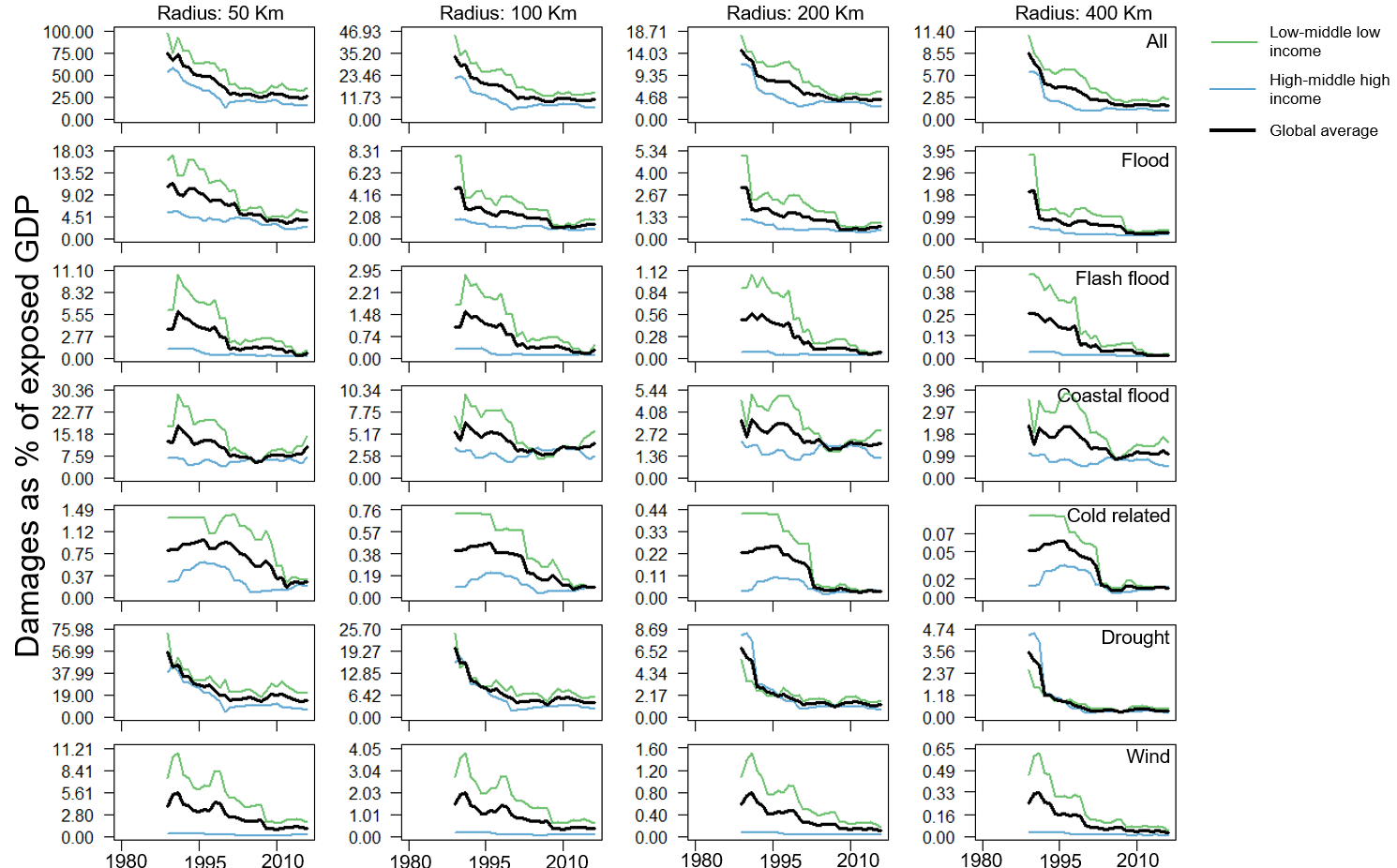


Figure A.4: Loss rates caused by the analyzed hazards splitting ratio of 50% vs 50%. Results are presented by income levels (low income in orange and high income in blue) and as global average (black).

**Appendix B**

Table B.1: Global average (over both income groups) mortality rate ratios between the periods 1980-1989 and 2007-2016

| Ratios period 1980-1989 to period 2007-2016 | | | | | |
| --- | --- | --- | --- | --- | --- |
| Analyzed Hazard | Radius 50 km | Radius 100 km | Radius 200 km | Radius 400 km | Average across radii |
| All hazards | 9.55 | 5.97 | 5.78 | 4.77 | 6.52 |
| General flood | 2.17 | 2.08 | 2.41 | 1.61 | 2.07 |
| Flash Flood | 32.45 | 15.29 | 10.51 | 8.93 | 16.79 |
| Coastal Flood | 7.47 | 6.61 | 6.11 | 5.66 | 6.46 |
| Cold Related | 4.97 | 3.60 | 3.78 | 2.70 | 3.76 |
| Heatwave | 1.43 | 1.31 | 4.49 | 1.71 | 2.23 |
| Wind | 8.83 | 6.93 | 7.37 | 8.03 | 7.79 |

Table B.2: Global average (over both income groups) loss rate ratios between the periods 1980-1989 and 2007-2016

| Ratios period 1980-1989 to period 2007-2016 | | | | | |
| --- | --- | --- | --- | --- | --- |
| Analyzed Hazard | Radius 50 km | Radius 100 km | Radius 200 km | Radius 400 km | Average across radii |
| All hazards | 3.37 | 3.57 | 5.06 | 7.01 | 4.75 |
| General flood | 2.66 | 3.19 | 3.95 | 6.29 | 4.02 |
| Flash Flood | 5.73 | 3.95 | 5.97 | 11.18 | 6.71 |
| Coastal Flood | 1.62 | 1.65 | 2.01 | 2.41 | 1.92 |
| Cold Related | 2.63 | 4.07 | 7.25 | 4.74 | 4.67 |
| Heatwave | 4.43 | 5.09 | 6.51 | 11.03 | 6.77 |
| Wind | 3.12 | 3.46 | 4.66 | 6.38 | 4.41 |

Table B.3: Mortality rate ratios low/middle low to high/middle high income for the period 1980-1989

| Ratios low/middle low to high/middle high income, period 1980-1989 | | | | | |
| --- | --- | --- | --- | --- | --- |
| Analyzed Hazard | Radius 50 km | Radius 100 km | Radius 200 km | Radius 400 km | Average across radii |
| All hazards | 15.27 | 10.14 | 7.51 | 8.49 | 10.4 |
| General flood | 4.00 | 2.05 | 1.80 | 1.50 | 2.3 |
| Flash Flood | 17.49 | 9.95 | 4.43 | 3.56 | 8.9 |
| Coastal Flood | 38.50 | 22.18 | 19.03 | 23.33 | 25.8 |
| Cold Related | 4.30 | 2.90 | 2.19 | 3.26 | 3.2 |
| Heatwave | 0.06 | 0.08 | 0.05 | 0.06 | 0.1 |
| Wind | 27.27 | 23.69 | 17.54 | 19.20 | 21.9 |

Table B.4: Mortality rate ratios low/middle low to high/middle high income for the period 2007-2016

| Ratios low/middle low to high/middle high income, period 2007-2016 | | | | | |
| --- | --- | --- | --- | --- | --- |
| Analyzed Hazard | Radius 50 km | Radius 100 km | Radius 200 km | Radius 400 km | Average across radii |
| All hazards | 4.80 | 4.47 | 4.17 | 4.23 | 4.4 |
| General flood | 1.54 | 1.52 | 1.30 | 1.45 | 1.5 |
| Flash Flood | 1.47 | 1.90 | 1.45 | 2.09 | 1.7 |
| Coastal Flood | 9.25 | 9.44 | 8.63 | 9.95 | 9.3 |
| Cold Related | 5.66 | 3.57 | 3.34 | 3.72 | 4.1 |
| Heatwave | 0.46 | 0.58 | 1.38 | 0.55 | 0.7 |
| Wind | 10.41 | 9.79 | 8.93 | 7.62 | 9.2 |

Table B.5: Loss rate ratios low/middle low to high/middle high income for the period 1980-1989

| Ratios low/middle low to high/middle high income, period 1980-1989 | | | | | |
| --- | --- | --- | --- | --- | --- |
| Analyzed Hazard | Radius 50 km | Radius 100 km | Radius 200 km | Radius 400 km | Average across radii |
| All hazards | 7.03 | 6.72 | 7.95 | 7.99 | 7.4 |
| General flood | 3.07 | 4.37 | 4.34 | 7.45 | 4.8 |
| Flash Flood | 5.25 | 5.62 | 10.96 | 13.73 | 8.9 |
| Coastal Flood | 4.29 | 3.11 | 3.46 | 4.16 | 3.8 |
| Cold Related | 4.97 | 8.13 | 13.48 | 7.35 | 8.5 |
| Drought | 1.38 | 1.17 | 0.56 | 0.47 | 0.9 |
| Wind | 23.24 | 17.95 | 14.91 | 14.79 | 17.7 |

Table B.6: Loss rate ratios low/middle low to high/middle high income for the period 2007-2016

| Ratios low/middle low to high/middle high income, period 2007-2016 | | | | | |
| --- | --- | --- | --- | --- | --- |
| Analyzed Hazard | Radius 50 km | Radius 100 km | Radius 200 km | Radius 400 km | Average across radii |
| All hazards | 4.58 | 3.85 | 3.14 | 3.05 | 3.7 |
| General flood | 2.57 | 2.08 | 2.06 | 2.02 | 2.2 |
| Flash Flood | 3.41 | 2.71 | 1.67 | 1.46 | 2.3 |
| Coastal Flood | 3.20 | 3.10 | 3.17 | 3.32 | 3.2 |
| Cold Related | 1.73 | 1.34 | 1.12 | 1.21 | 1.4 |
| Drought | 3.10 | 2.37 | 1.90 | 1.80 | 2.3 |
| Wind | 13.50 | 11.50 | 8.90 | 8.50 | 10.6 |

**Appendix C**


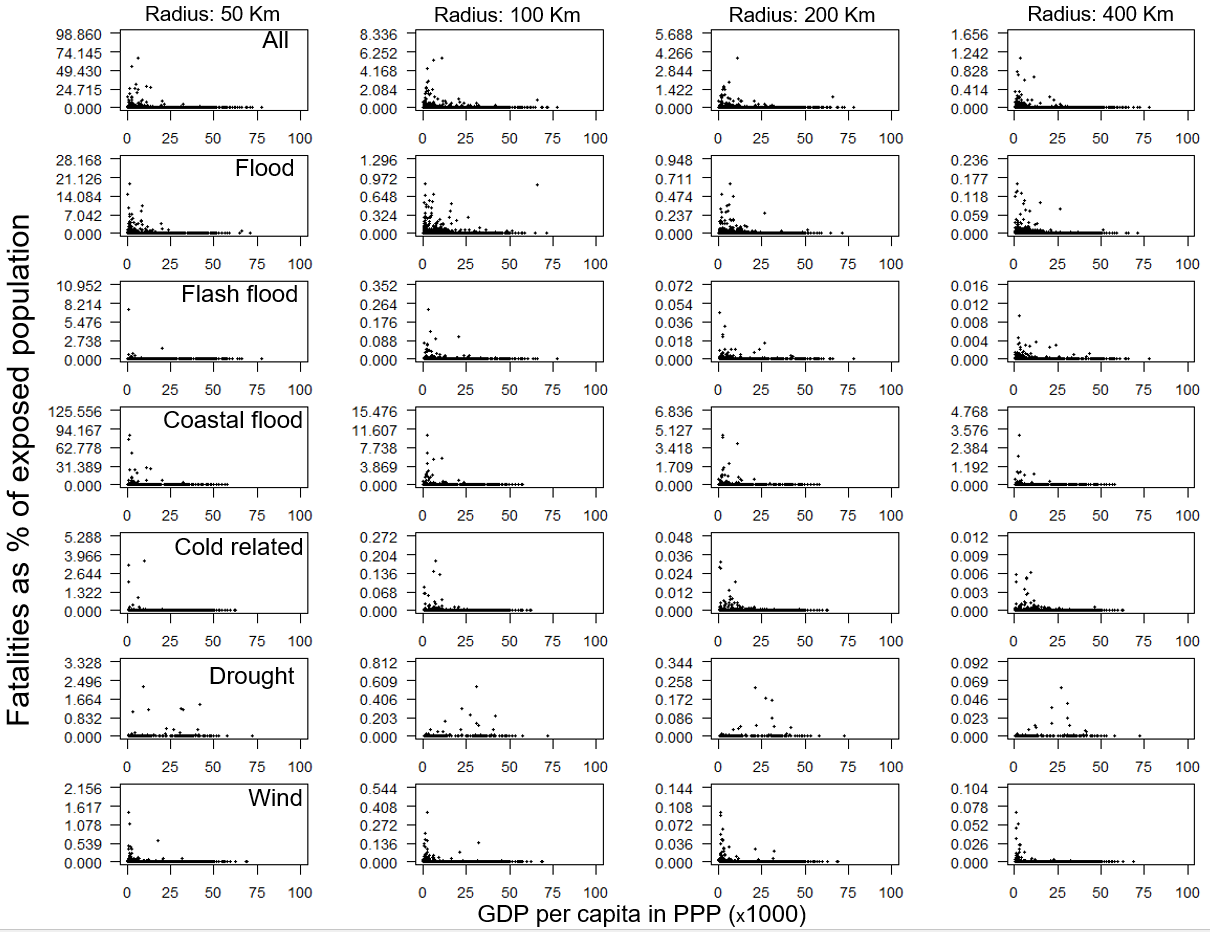


Figure C.1: Morality rate vs GDP per capita (in US$-PPP x1000) in the country at the time of the reported event.


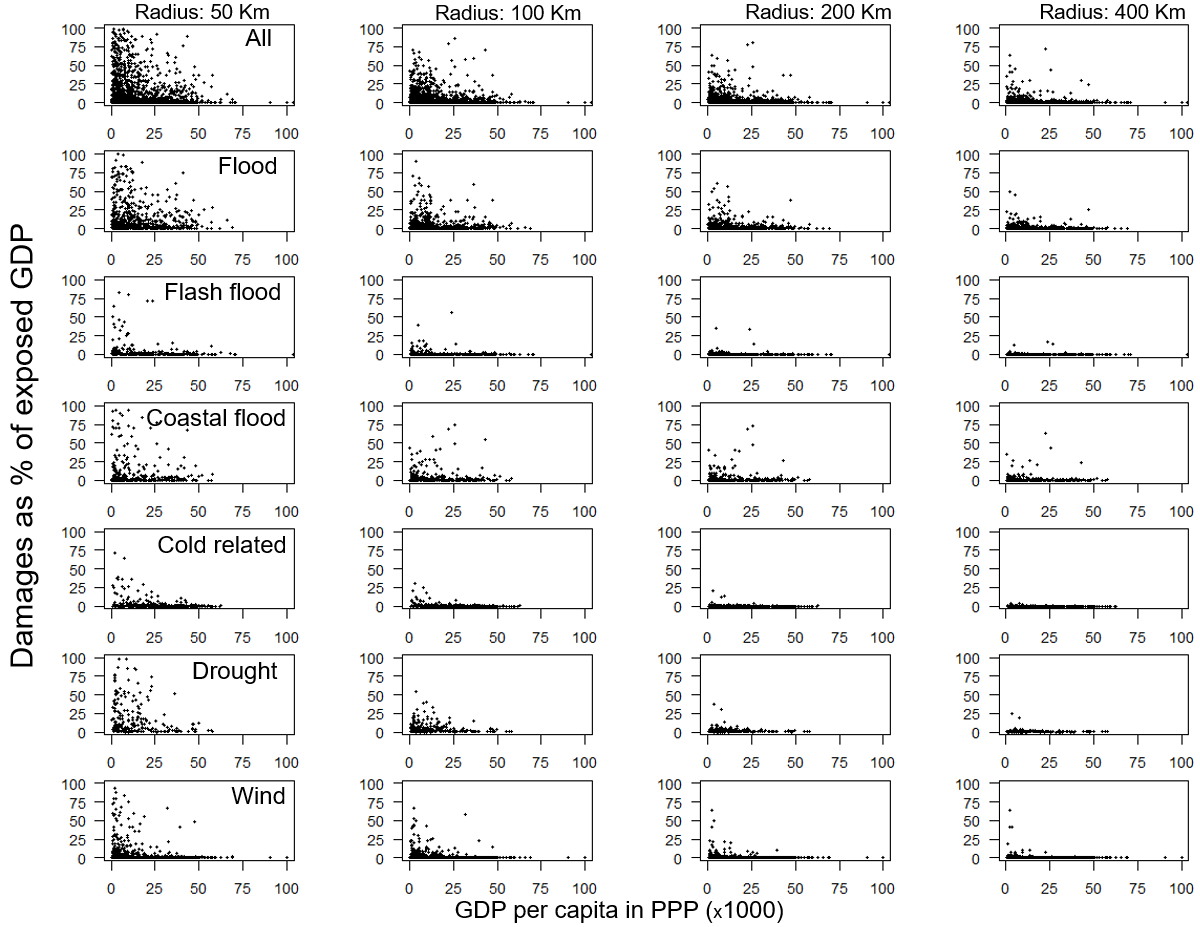
Figure C.2: Loss ratio vs GDP per capita (in US$-PPP x1000) in the country at the time of the reported event.

**Appendix D**


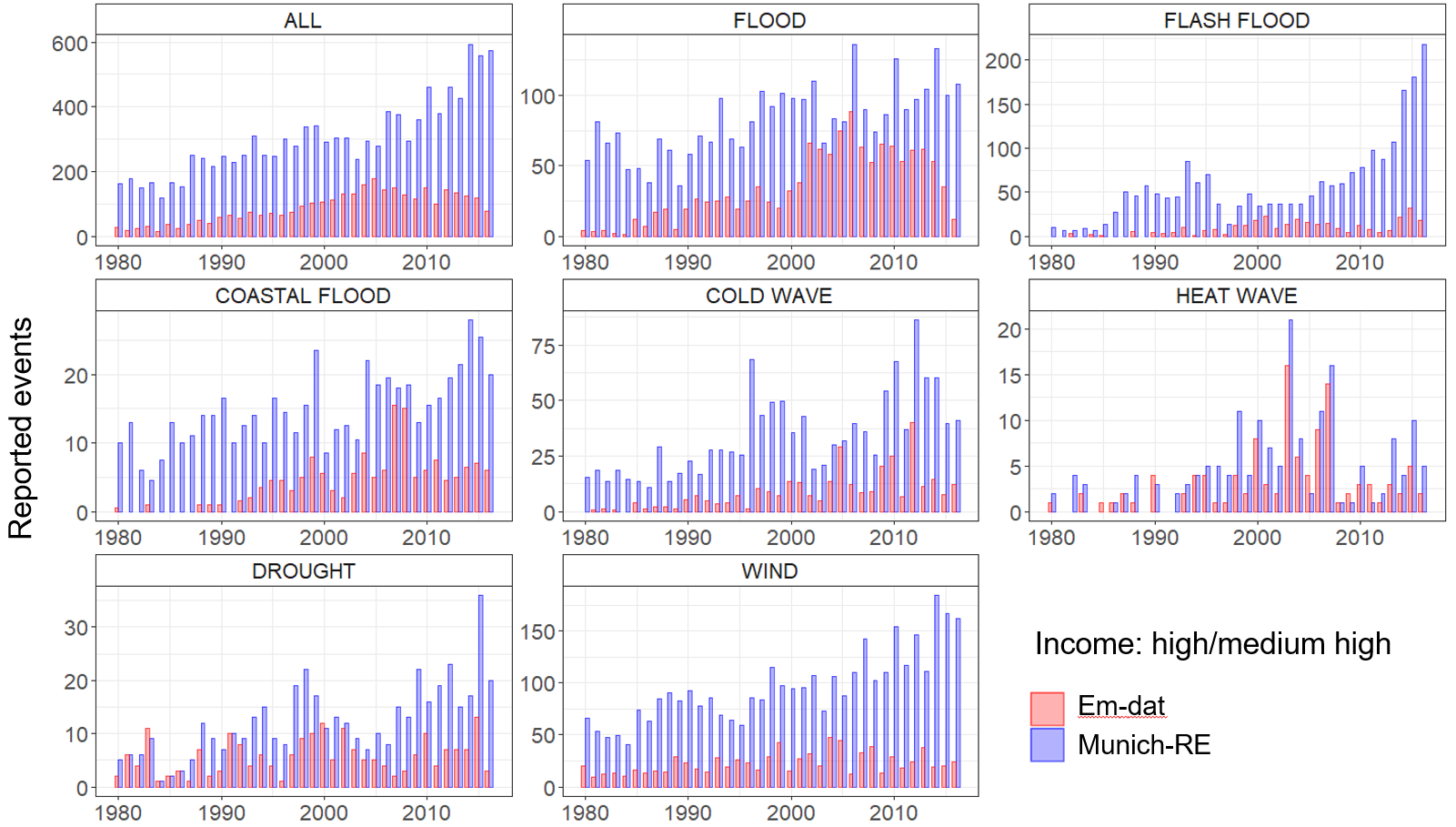


Figure D.1: Reported events in NatCatSERVICE and EM-DAT for high/medium high income countries.


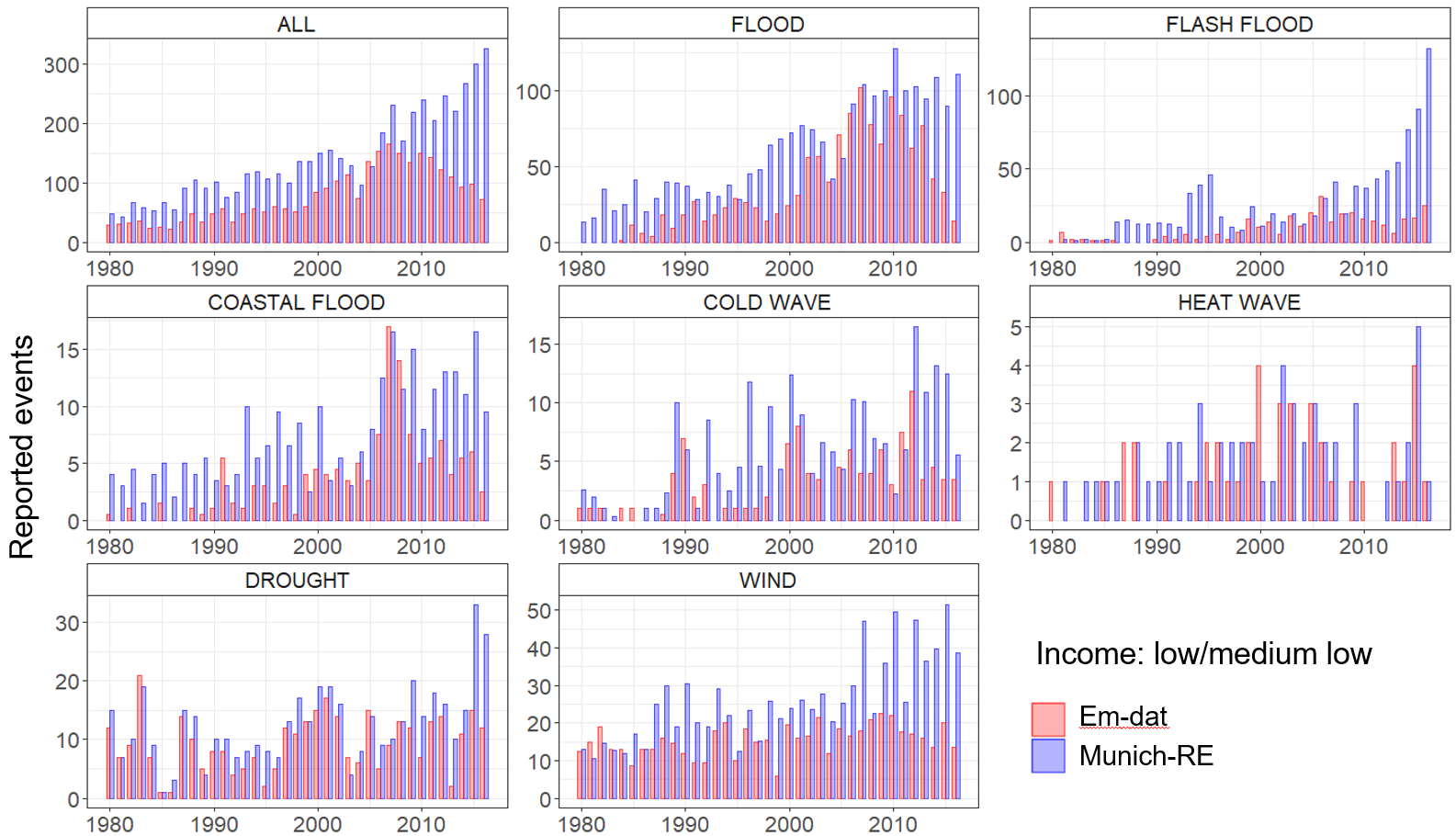


Figure D.2: Reported events in NatCatSERVICE and EM-DAT for low/medium low income countries.


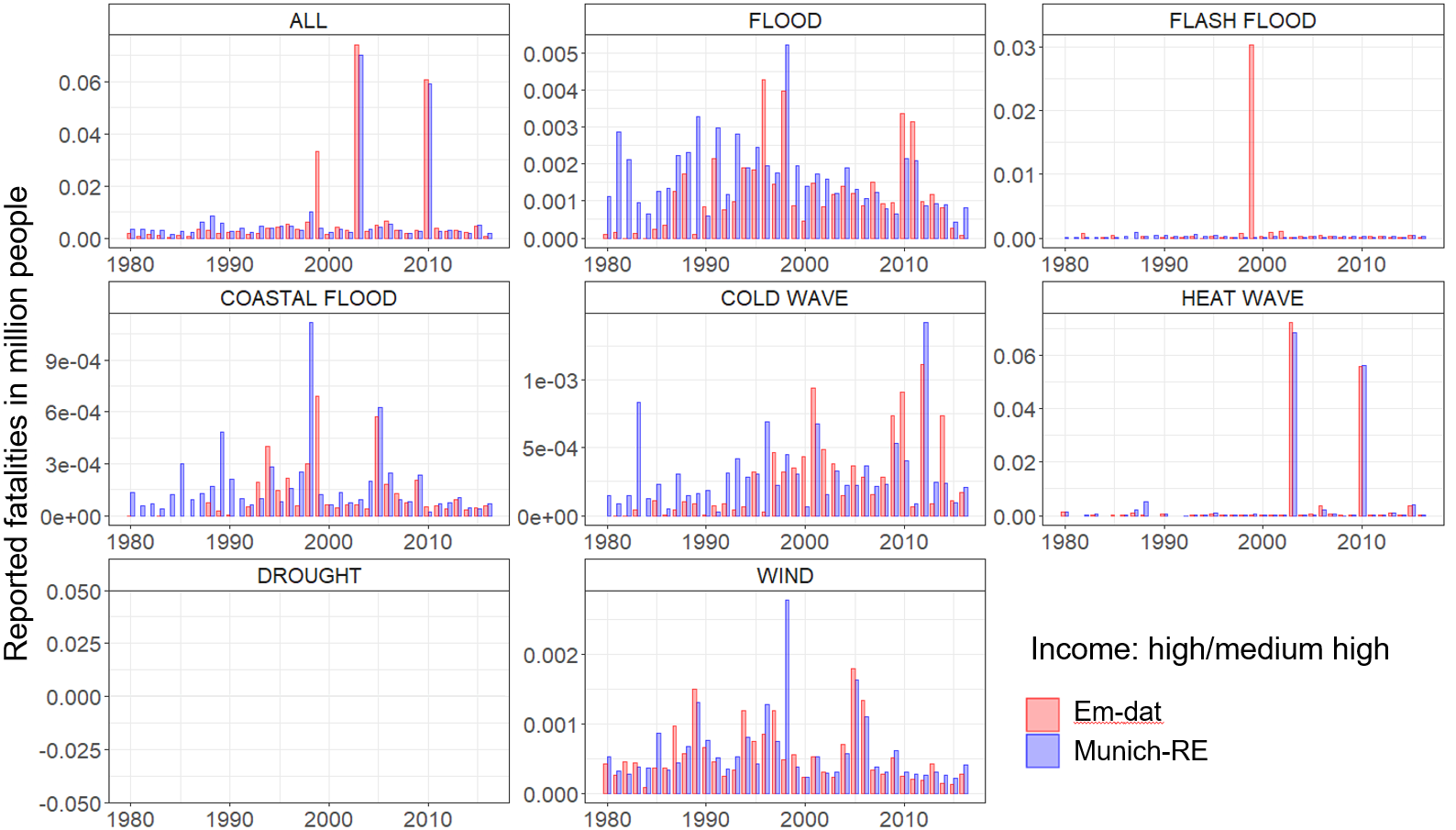


Figure D.3: Reported fatalities in NatCatSERVICE and EM-DAT for high/medium high income countries.


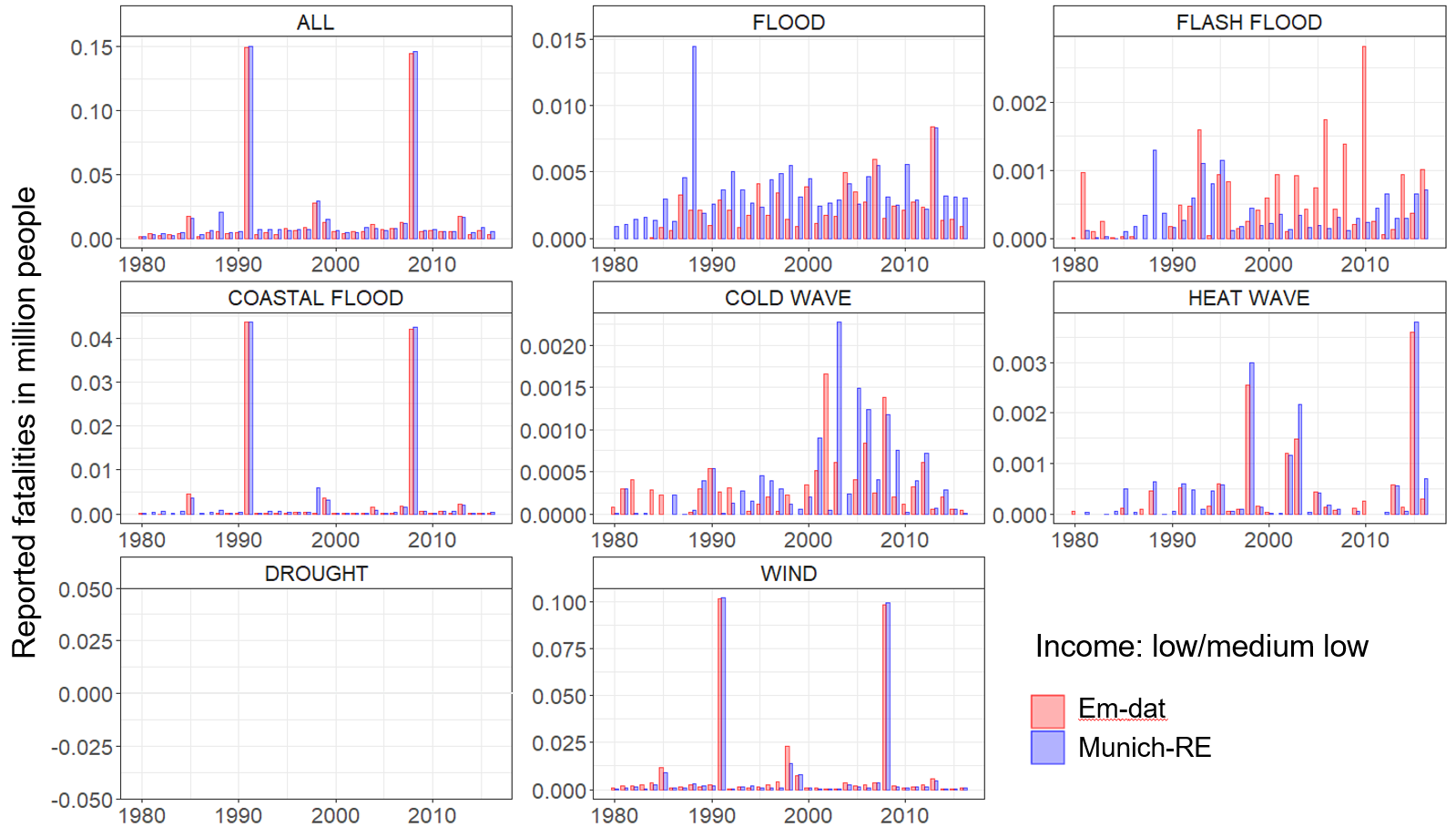


Figure D.4: Reported fatalities in NatCatSERVICE and EM-DAT for low/medium low income countries.


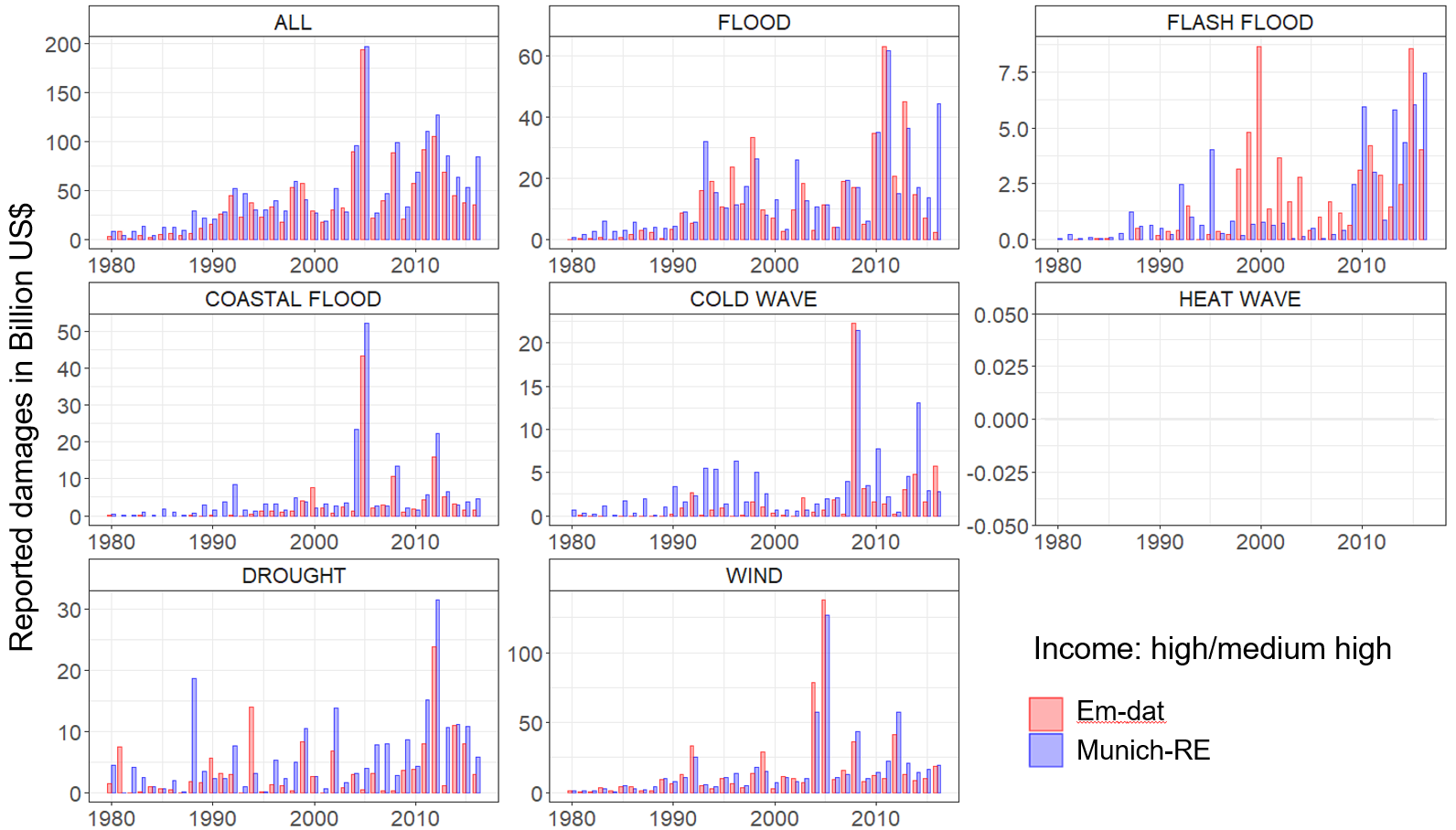


Figure D.5: Reported damages in NatCatSERVICE and EM-DAT for low/medium low income countries.


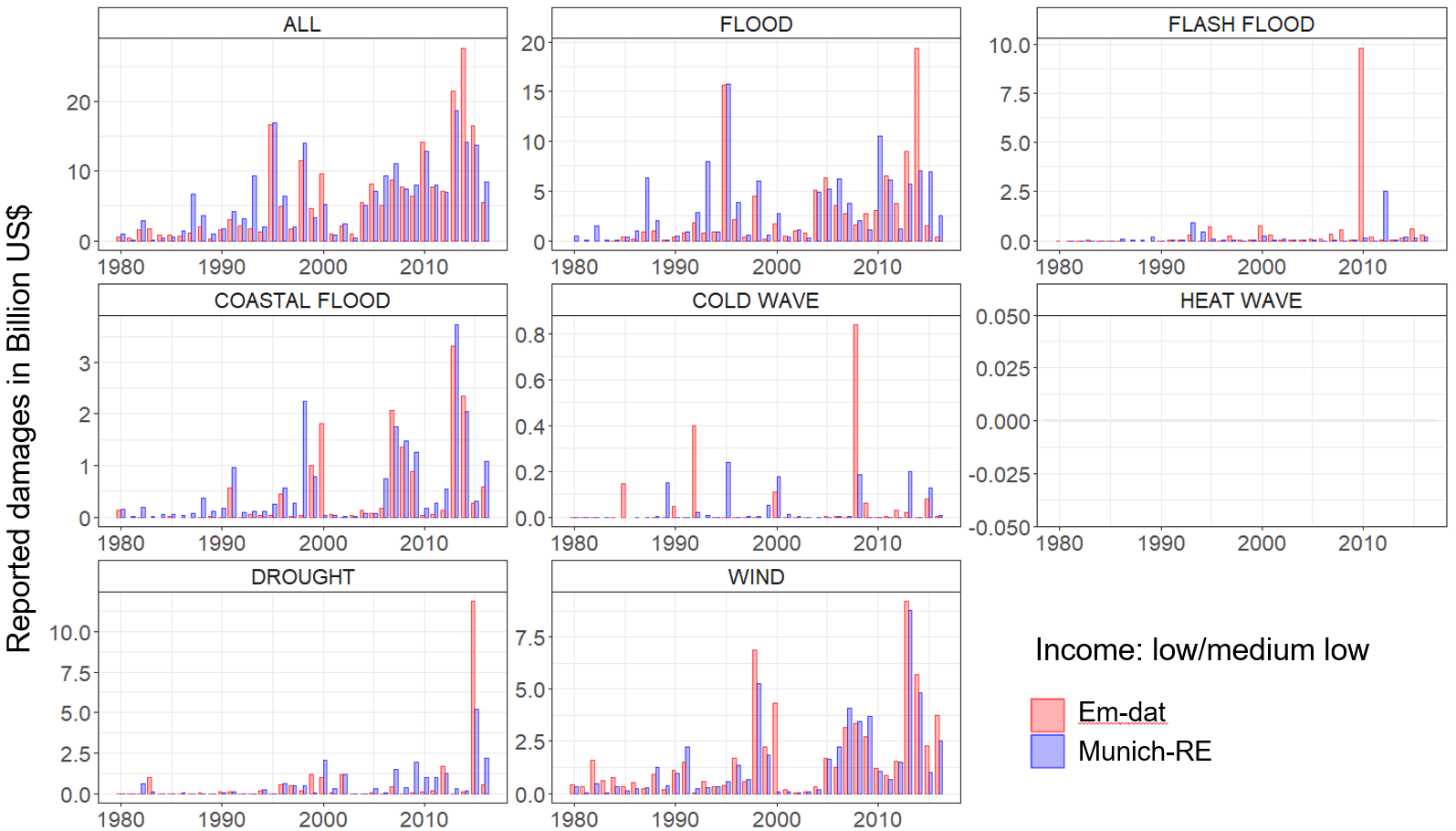


Figure D.6: Reported damages in NatCatSERVICE and EM-DAT for low/medium low income countries.
